# Sustainable Triacetic Acid Lactone Production from Sugarcane by Fermentation and Crystallization

**DOI:** 10.1021/acssuschemeng.5c04797

**Published:** 2025-10-16

**Authors:** Sarang S. Bhagwat, Marco Nazareno Dell’Anna, Yalin Li, Mingfeng Cao, Emma C. Brace, Sunil S. Bhagwat, George W. Huber, Huimin Zhao, Jeremy S. Guest

**Affiliations:** † DOE Center for Advanced Bioenergy and Bioproducts Innovation (CABBI), 124331 University of Illinois Urbana-Champaign, 1206 W. Gregory Drive, Urbana, Illinois 61801, United States; ‡ The Grainger College of Engineering, Department of Civil and Environmental Engineering, University of Illinois Urbana-Champaign, 3221 Newmark Civil Engineering Laboratory, 205 N. Mathews Avenue, Urbana, Illinois 61801, United States; § Department of Chemical and Biological Engineering, 5228 University of Wisconsin-Madison, 1415 Engineering Drive, Madison, Wisconsin 53706, United States; ∥ Department of Civil and Environmental Engineering, 242612 Rutgers, The State University of New Jersey, 500 Bartholomew Rd, Piscataway, New Jersey 08854, United States; ⊥ Department of Chemical and Biomolecular Engineering, University of Illinois Urban-Champaign, 215 Roger Adams Laboratory, 600 S. Mathews Avenue, Urbana, Illinois 61801, United States; # Department of Engineering, 6019 Boston College, 245 Beacon St, Chestnut Hill, Massachusetts 02467, United States; ∇ Department of Chemical Engineering, Institute of Chemical Technology, Nathalal Parekh Marg, Matunga, Mumbai, Maharashtra 400019, India; ○ Department of Chemistry, Indian Institute of Science Education and Research (IISER) Pune, Dr. Homi Bhabha Road, Pune, Maharashtra 411008, India; ◆ Institute for Sustainability, Energy, and Environment (iSEE), University of Illinois Urbana-Champaign, 1101 W. Peabody Drive, Urbana, Illinois 61801, United States

**Keywords:** biorefinery design and sizing, techno-economic analysis (TEA), life cycle assessment (LCA), titer−yield opportunity space, uncertainty, financial viability, greenhouse gas emissions, sweet sorghum, triacetic acid lactone (TAL) solubility

## Abstract

Triacetic acid lactone (TAL) has the potential to serve as a bioderived platform chemical for commercial products including sorbic acid and recyclable polydiketoenamine plastics. In this study, we leveraged BioSTEAM to design, simulate, and evaluate (via techno-economic analysis, TEA, and life cycle assessment, LCA) TAL production from sugarcane. We experimentally characterized TAL solubility, calibrated solubility models, and designed a process to separate TAL from fermentation broths by crystallization. The biorefinery could produce TAL at a minimum product selling price (MPSP) of $3.73–5.86·kg^–1^ (5th–95th percentiles; baseline at $4.60·kg^–1^) and a carbon intensity (CI) of 5.31 [2.60–8.71] kg CO_2_-eq·kg^–1^, which could enable financially viable, low-CI production of sorbic acid and polydiketoenamines. To drive down costs and CI, we explored the theoretical fermentation space (titer, yield, productivity combinations), operation scheduling and capacity expansion strategies (e.g., integrated sorghum processing), and potential separation improvements (mitigating TAL loss through pH control). Advancements in key design and technological parameters could further reduce MPSP by 51% to $2.26·kg^–1^ [$1.97–2.80·kg^–1^] and CI by 43% to 3.05 [1.91–4.15] kg CO_2_-eq·kg^–1^. This research highlights the ability of agile TEA-LCA to screen promising designs, navigate sustainability trade-offs, prioritize research needs, and chart quantitative roadmaps to advance bioproducts and biofuels.

## Graphical abstract

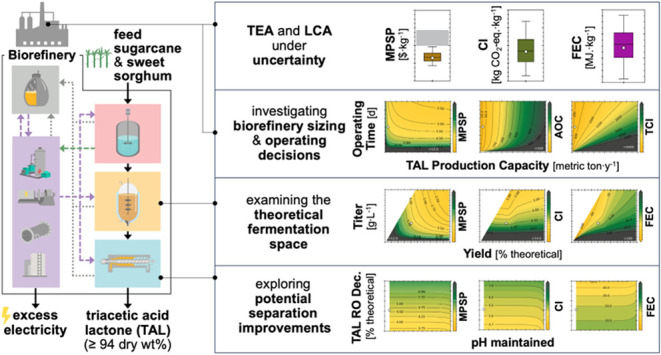

## Introduction

Triacetic acid lactone (TAL) has been identified as a bioprivileged chemical—a bioderived chemical intermediate that can be converted into a diverse set of useful chemical products.^1–5^ This utility has motivated recent efforts to investigate the potential use of TAL as a platform chemical in the production of commercially important commodity chemicals (e.g., sorbic acid and potassium sorbate,^2,6^ acetylacetone^2^), specialty chemicals (e.g., pogostone,^7^ katsumadain,^8^ penicipyrone^9^), and novel chemicals with the ability to serve as functional replacements to existing products (e.g., highly recyclable and thermally stable polydiketoenamine plastics,^10^ enhanced corrosion inhibitors in steel equipment^3^). In 2019, an estimated 72,350 metric tons of sorbic acid (with a market value of $480 million) was consumed globally, and this demand is projected to grow at 3.8% annually from 2020–2030 (to approximately 104,000 metric tons·y^–1^, with a market value of $770 million) largely due to its increasing usage as a preservative in the food and beverage industry.^11^ A more recent report estimated a global sorbic acid market of 150,000 metric tons in 2023 and projected a 4.8% annual growth to 260,000 metric tons·y^–1^ by 2034.^12^

Currently, both TAL and sorbic acid are produced almost exclusively via chemical synthesis.^5,11,13^ Sorbic acid is primarily produced via the condensation of malonic acid and crotonaldehyde,^4,13^ which are both primarily fossil-derived chemicals.^14–16^ Alternatively, sorbic acid can be produced from TAL through a series of reactions (namely hydrogenation, dehydration, ring-opening, and hydrolysis) with high overall yields (e.g., approximately 77% as potassium sorbate).^2,6^ TAL does not currently have an established global market as the chemical synthesis route is prohibitively expensive.^5^ However, the prospects for the biological production of TAL continue to improve, with recent advancements in the conversion of sugars and acetate by metabolically engineered strains of microbes including *Saccharomyces cerevisiae*,^17–21^ *Yarrowia lipolytica*,^7,22–25^ *Escherichia coli*,^10,20,26^ and *Rhodotorula toruloides* ^27^ (formerly classified as *Rhodosporidium toruloides* ^28^). By integrating the biological production of TAL with catalytic upgrading to sorbic acid, we have the potential to produce bioderived sorbic acid with greater financial viability and environmental benefits than conventional, fossil-derived production.

To achieve biological production of TAL at the industrial scale, key challenges related to fermentation and separation of TAL from the fermentation broth need to be overcome. In particular, the poor performance of fermentation microbes results in high costs associated with feedstock acquisition (due to low yield) and product separation (due to low titer).^10,29^ To overcome low titers, a recently proposed separation process leveraged activated carbon for adsorption of TAL from the fermentation broth with 72% recovery, but this process may be undermined by biologically derived impurities due to nonselective adsorption.^29^ Crystallization has been suggested as an alternative method for low-cost separation of TAL.^10^ However, the high cell density associated with TAL production (e.g., up to 47 g cell mass·L^–1^ broth^25^) coupled with the low solubility of TAL in water at fermentation operating temperatures (e.g., 8.41 g·L^–1^ at 30 °C;^30^ the temperature maintained for TAL production by *Y. lipolytica* is 28–30 °C^7,22–25^ ) poses difficulties for selective TAL recovery by crystallization. If insoluble solids (cell mass and crystallized TAL) were directly centrifuged out of the broth, it may be difficult to obtain a pure TAL stream free of cellular debris. Although it may be possible to separate TAL from fermentation broths through crystallization more effectively, design and simulation of such processes has been limited due to the lack of data and models for TAL solubility in water at relevant temperatures.

Further, despite the potential of biobased TAL as a platform chemical for sustainable biomanufacturing, we are only aware of three studies that characterized its financial viability (via techno-economic analysis, TEA)^10,29,31^ and one study that characterized its life cycle environmental impacts (via life cycle assessment, LCA).^10^ In these previous studies, a lack of available data and validated solubility models often (understandably) required authors to make simplifying assumptions. These necessary assumptions included neglecting the low solubility of TAL in aqueous solutions (assuming TAL was completely dissolved, even in high-titer fermentation broths),^31^ assuming a stable supply of sugar as the feedstock (without considering the impact of feedstock harvest schedules on biorefinery annual operating days),^29^ and assuming co-utilization of glucose and xylose based on fermentation performance observed in experimental work solely using glucose.^10^ Ultimately, prioritizing research and development for biobased TAL production would benefit from consideration of the end-to-end process with robust modeling under uncertainty and by evaluating the system sustainability implications of technological improvements beyond the current state-of-technology.

The objectives of this study were to evaluate the potential for sustainable production of TAL from renewable, sugar-based feedstocks across a landscape of technology performance scenarios, and guide future research and development pathways to advance biobased TAL production. To this end, we leveraged BioSTEAM,^32,33^ an open-source platform in Python, to assess the potential for the financially viable and environmentally sustainable production of TAL. First, we experimentally characterized TAL solubility in water at temperatures ranging from 0 to 93 °C and for the ring-opening decarboxylation of TAL in water to acetylacetone (Figure 1). Next, we fit thermodynamic solubility models to the obtained TAL solubility data and, using the calibrated solubility model, we designed a process to separate TAL from fermentation broths by crystallization. We developed a full biorefinery design to produce TAL from sugarcane and sweet sorghum and analyzed a baseline scenario with the microbial strain *Y. lipolytica* using demonstrated fermentation performance from the literature.^22^ We then performed Monte Carlo simulations to characterize the uncertainty in sustainability indicators (minimum product selling price, MPSP; life cycle carbon intensity, CI; fossil energy consumption, FEC) and sensitivity analyses to identify key sustainability drivers. To better understand the economic and environmental implications of potential process improvements, we designed and simulated biorefineries across the entire theoretical fermentation space (i.e., across all possible titer, yield, and productivity combinations). Further, we computationally explored the sustainability implications of potential strategies to mitigate the ring-opening decarboxylation of TAL by controlling the pH of the stream during heating (i.e., step 1 in Figure 2B) by adding a base, sodium hydroxide. In addition, we explored the economic implications of alternative biorefinery operating schedules (including the integration of sweet sorghum as an additional feedstock) and TAL production capacities. Finally, we discuss and prioritize research and development opportunities along the value chain to advance the financial viability and environmental sustainability of biobased TAL production.

**Figure 1:**
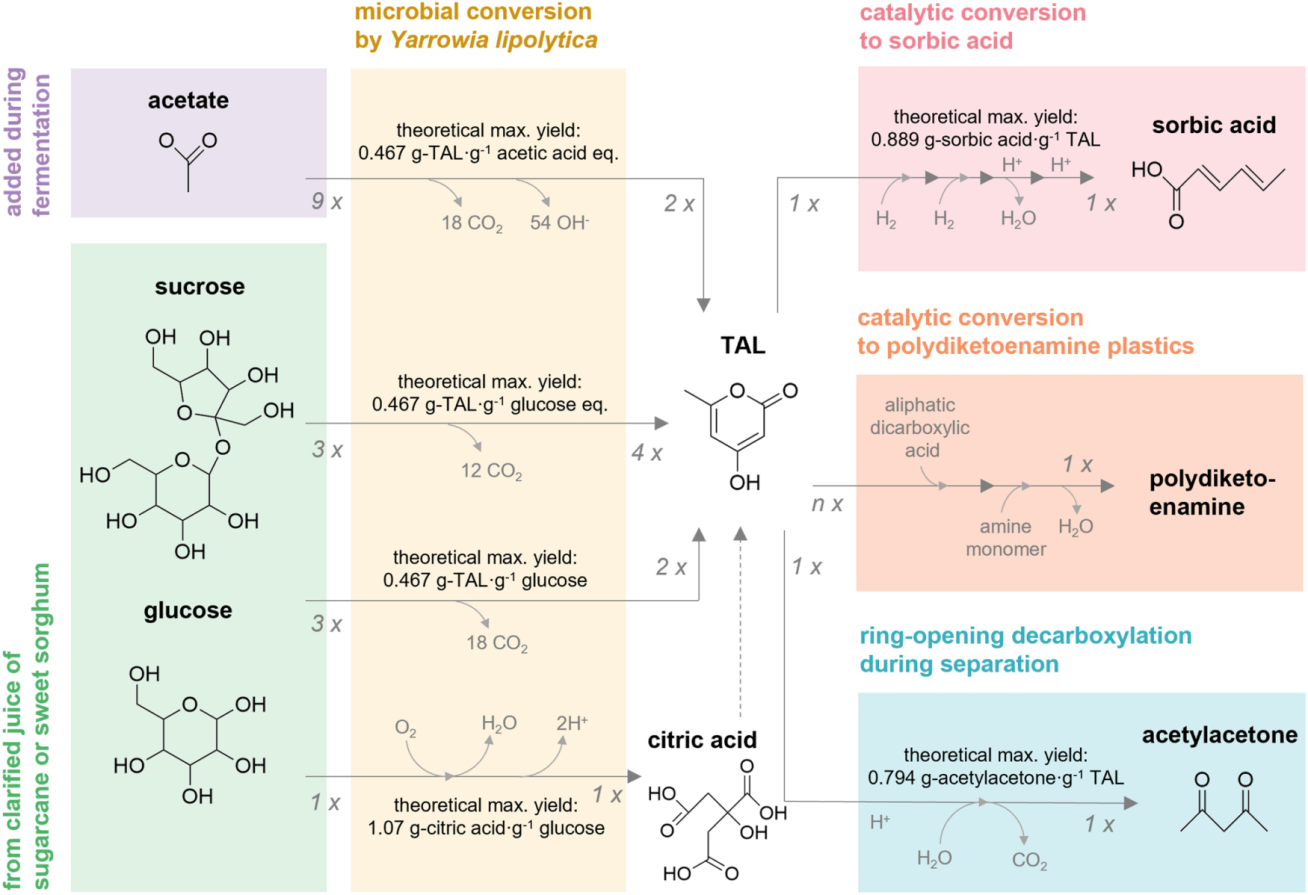
Overview of key reactions discussed in this work. Chemical structures are depicted immediately below the names of the compounds they represent. Numbers at beginnings and ends of arrows denote stoichiometric coefficients of reactants and products, respectively. Theoretical maximum yields by mass (*theoretical max. yield*) are shown for each reaction. Although citric acid may be used by *Y. lipolytica* for TAL production in the absence of glucose, this is not depicted because TAL production was modeled solely from sucrose, glucose, and xylose. Citric acid production from glucose was modeled based on the final yield after citric acid depletion for TAL production reported by Markham et al.^22^ Reaction intermediates are omitted for clarity. In the case of catalytic conversion of TAL to sorbic acid, intermediates (serially: 5,6-dihydro-4-hydroxy-6-methyl-2H-pyran-2-one; 4-hydroxy-6-methyltetrahydro-2-pyrone; and parasorbic acid) can occur in a series of reactors (e.g., for hydrogenation, dehydration and ring-opening, and hydrolysis, respectively).^2,6^ In the case of catalytic conversion of TAL to polydiketoenamine plastics, aliphatic dicarboxylic acids can be used along with TAL (stoichiometric coefficient *n* will depend on the structure of the targeted resin) to make biobased monomers, which can be milled with amine monomers (1:1 stoichiometry with TAL) to make polydiketoenamine resins (chemical structures are not depicted for clarity, and theoretical maximum yields will depend on the aliphatic dicarboxylic acids used).^10^

**Figure 2:**
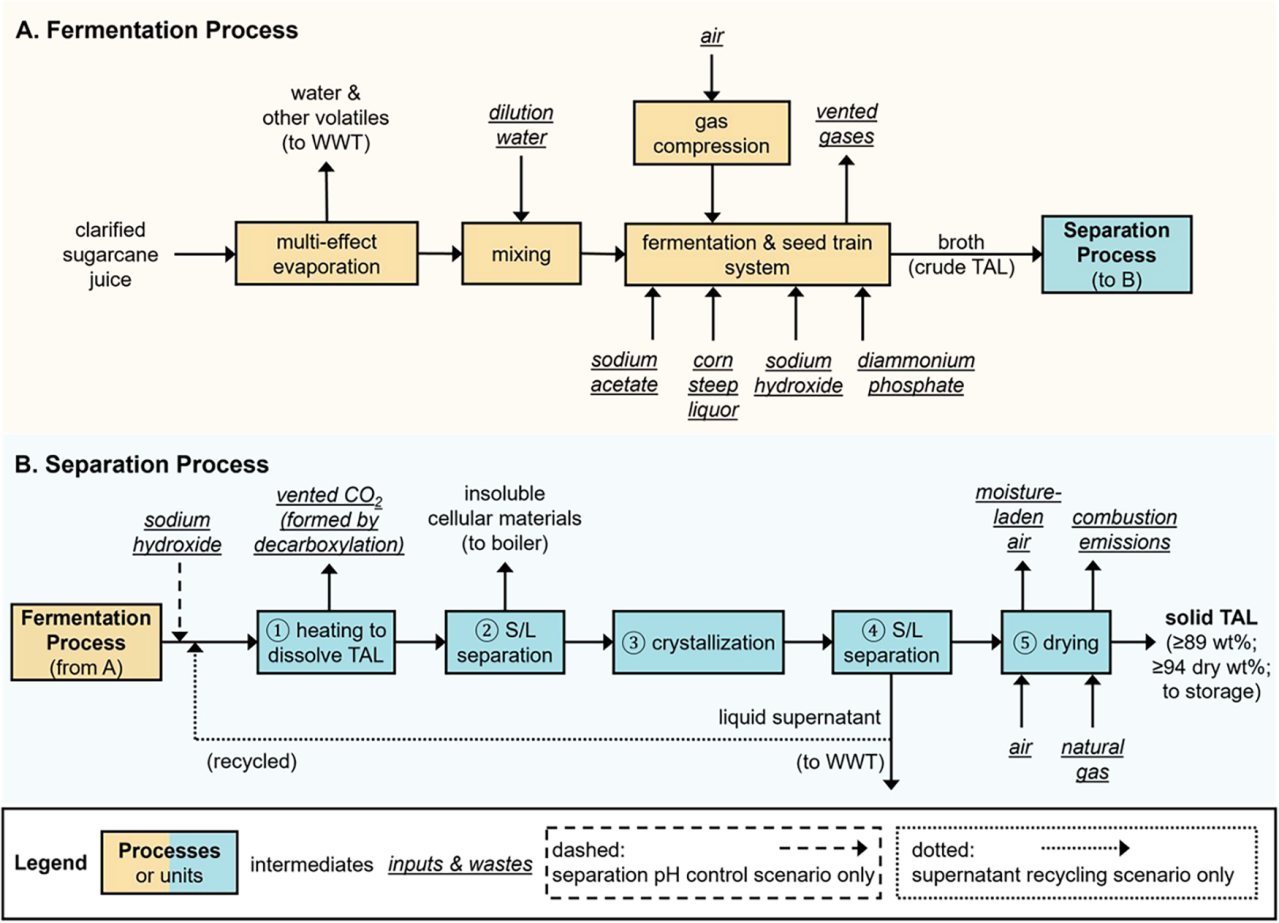
Simplified block flow diagram for the (A) fermentation and (B) separation processes. WWT denotes wastewater treatment. Some units (e.g., pumps, mixers, splitters, heat exchangers) are not included in the figure for clarity; the process flow diagram in the system report (available in the online repository^44^) includes the full set of details.

## Methods

### Estimating the Market Opportunity

Although TAL can be catalytically upgraded to a diverse set of specialty, commodity, and novel chemicals, it does not yet have an established global market. A TAL market price of $10·kg^–1^ has been previously suggested based on the potential for TAL to serve as a direct replacement for the petrochemical dimedone^10^ to synthesize polydiketoenamines—highly recyclable plastics.^34,35^ A recent study demonstrated biobased TAL can be used to produce polydiketoenamine plastics with greater thermal stability and a wider range of serviceable applications than the petrochemical dimedone.^10^ The estimated global demand for biobased plastics was approximately 1.05 million metric tons in 2023 and could grow 9.3% annually to 1.63 million metric tons by 2028,^36^ indicating a large market opportunity. As an alternative benchmark, TAL could serve as a feedstock for sorbic acid production. TAL can be upgraded to sorbic acid through a series of reactions (hydrogenation, dehydration, ring-opening, and hydrolysis) with a theoretical maximum yield of 0.889 g sorbic acid·g TAL^–1^. Sorbic acid is a commodity chemical with one report estimating a global demand of 72,350 metric tons in 2019 (expected to grow at 3.8% annually from 2020 to 2030).^11^ A more recent report actually estimated the 2023 global sorbic acid market was 150,000 metric tons and could grow 4.8% annually to 260,000 metric tons·y^–1^ by 2034.^12^ Due to its antimicrobial properties, sorbic acid is mainly used as a preservative in foods and beverages, pharmaceuticals, and animal feed.^11^ It is also used as a preservative in cosmetics, biomanufacturing processes, and in formulations for soaps and detergents.^11^ The U.S. ranks first globally in sorbic acid consumption (about 23,800 metric tons in 2019) and produces roughly 50% of this amount, also relying on imports to satisfy the demand.^11^ The 2019 market price of sorbic acid was $6.74·kg^–1^ in the U.S, and a 2023 search for vendor listings on Alibaba (with the “verified” and “trade assurance” filters active) of bulk sorbic acid orders showed a lowest selling price listed as potassium sorbate at $6.50·kg^–1^ potassium sorbate (equivalent to $8.71·kg^–1^ sorbic acid assuming 100% conversion).^11,37^ Based on the theoretical maximum yield of sorbic acid from TAL, it follows that the TAL price must be below $5.99–7.74·kg^–1^ to have any potential for market-competitive sorbic acid production. This price range neglects costs associated with TAL conversion to sorbic acid, but also neglects potential financial incentives for bioderived products (e.g., government incentives,^38^ consumers’ willingness to pay higher prices^39^). Thus, to inform the discussion of TAL financial viability in this work, we benchmarked TAL MPSP results against the range of $5.99–7.74·kg^–1^ (for TAL as a feedstock to produce sorbic acid) and the aforementioned literature value of $10·kg^–1^ (for TAL to replace dimedone as a feedstock to produce polydiketoenamine plastics^10^).

### System Description

#### Juicing, Fermentation, and Separation Processes

The biorefineries in this study are comprised of three main (inside battery limits) processes (feedstock juicing and clarification, fermentation, and separation) with outside-battery wastewater treatment and miscellaneous facilities (a biorefinery overview is provided in Figure S1, and a detailed list of biorefinery equipment is provided in Table S2 in the Supporting Information, SI). The biorefinery’s production capacity was 13,385 metric tons TAL·y^–1^ in the baseline case, which would be enough to produce 11,900 metric tons of sorbic acid annually assuming theoretical maximum conversion. This production capacity was chosen based on (i) the growth projected in the annual U.S. demand for sorbic acid between 2020–2030 (from approximately 23,800 metric tons of sorbic acid in 2019 to a projected 34,550 metric tons of sorbic acid in 2030) and (ii) the amount by which the 2019 U.S. consumption of sorbic acid (23,800 metric tons) exceeded the 2019 U.S. production capacity (12,375 metric tons·y^–1^).^11^ This translates to a baseline biorefinery accepting 620,540 metric tons·y^–1^ sugarcane, which is well within the reported annual capacity for an intermediate-size sugarcane processing facility (1,600,000 metric tons^40^) that has been assumed in previous sugarcane biorefinery TEAs.^41–43^ Larger production capacities were also explored to improve financial viability (Section S1.6 of the SI). The biorefinery was assumed to operate 180 days annually in the baseline case, an operating time previously estimated for sugarcane biorefineries in the southern U.S. based on typical harvest periods and maximum storage times.^41–43^ Assumptions related to feedstock composition (Table S1) are detailed in the SI. In the sugarcane juicing and clarification system, the oilcane is crushed, and the extruded juice is treated and filtered to remove impurities. The process models used for sugarcane juicing and clarification are described in previous studies.^33,41^

The bagasse from crushing feed sugarcane is diverted to the boiler for combustion and the clarified juice is sent to the fermentation process. In the fermentation process, the juice undergoes either multiple-effect evaporation or dilution as needed to achieve the necessary concentration of sugars (Figure 2A). The evaporated or diluted juice is sent to fermentation with *Y. lipolytica*. Note that, to be consistent with chemical engineering literature, the term *fermentation* is used here to mean microbial conversion (including aerobic conversion) of a substrate to a specific product in a bioreactor. Sodium acetate is also fed into the fermentation reactor as an additional carbon source for TAL production. In addition, corn steep liquor and diammonium phosphate were added to the fermentation broth to satisfy microbial nitrogen and phosphorus requirements, respectively (further explained in Section S1.1 in the SI), and sodium hydroxide is fed to maintain a pH of 6.5 (further explained in Sections S1.1 and S1.4 in the SI). Based on data from the literature, the baseline fermentation performance was assumed to achieve an overall TAL yield of 40.5% of the theoretical maximum yield on glucose and acetate (the values of the theoretical maximum yields on glucose and acetate being approximately equal at 0.467 g-TAL·g-glucose eq^–1^ and 0.467 g-TAL·g-acetic acid eq^–1^, respectively^22^), a maximum titer of 35.9 g·L^–1^, and a productivity of 0.12 g·L^–1^·h^–1^ (reasoning provided in Section S1.1 and Table S3 in the SI). Some glucose was assumed to be converted to citric acid with a yield of 0.094 g·g^–1^ based on the reported concentrations in the fermentation media and broth.^22^ Additional fermentation design and operational details are provided in Section S1.1 and baseline values and distributions for all process parameters included in the uncertainty analysis are listed in Table S6 in the SI.

After fermentation, the produced broth containing TAL, insoluble cellular materials, and other impurities is directed to the separation process. The design of this separation process was enabled by the experimentally calibrated temperature-dependent solubility model for TAL (discussed in Results and Discussion). First, the broth is heated to a sufficiently high temperature to dissolve all TAL present (step 1 in Figure 2B), after which insoluble cellular materials are centrifuged out (step 2 in Figure 2B) and the liquid effluent is sent to crystallization at 1 °C (step 3 in Figure 2B). A second centrifugation unit (step 4 in Figure 2B) separates the supernatant, which is diverted to wastewater treatment, from the crystallized TAL, which is dried (step 5 in Figure 2B) and sent to storage. Based on experimental observations while heating TAL in water (Section S1.2 in the SI), we modeled ring-opening decarboxylation of TAL to 2,4-pentanedione (acetylacetone), which was assumed to remain in the liquid supernatant due to its low melting point (−23 °C^45^) and high solubility in water (e.g., 160 g·L^–1^ at 25 °C^45^). Evaporation of water from the broth before crystallization was not considered as heating TAL in aqueous solutions can result in TAL loss by ring-opening decarboxylation^2,46^ (a detailed discussion of conditions favoring TAL ring-opening decarboxylation is included in Section S2.5 of the SI). In addition to the baseline separation process, we also explored the possibility of mitigating ring-opening decarboxylation of TAL through pH control by simulating adding purchased sodium hydroxide prior to heating (step 1 in Figure 2B; discussed in Section S1.7 of the SI), and the possibility of recycling the supernatant for improved recovery (Figure S2; discussed in Section S2.1 of the SI).

#### Thermodynamic Modeling of TAL Solubility in Water as a Function of Temperature

TAL solubility in water was experimentally measured at temperatures ranging from 0 °C–93 °C (Section S1.2 in the SI). Further, we observed ring-opening decarboxylation of the dissolved TAL to 2,4-pentanedione (acetylacetone) to occur when heating the solution—a phenomenon previously reported in the literature^2,46^ —and we specifically measured the TAL ring-opening decarboxylation conversion at temperatures ranging from 30 °C–80 °C (Section S1.2 in the SI). We modeled the solubility of TAL in water using the equation provided by Poling, Prausnitz, and O’Connell^47^ for solid solutes, with the solute activity coefficient modeled using a one-parameter van Laar equation by applying the parameter reduction method suggested by Poling, Prausnitz, and O’Connell^47^ (eq S3 in the SI; discussed in detail in Section S1.3 of the SI).

#### Facilities

Facilities in the biorefinery include a boiler (for on-site heat utility production), turbogenerator (for on-site electricity production), a cooling tower and chilled water system (for on-site cooling utility production), wastewater treatment (using a newly developed high-rate process scheme that includes internal circulation reactors and anaerobic membrane bioreactors for biogas production^48^), heat exchanger network (HXN, for heat integration to minimize heating and cooling utility demands), process water center (for water reuse), and other auxiliary units for storage, air distribution, and clean-in-place. These facilities were modeled to be consistent with previous studies (additional details presented in Section S1.7 in the SI).^49–51^

#### Open-Source System Model

The biorefinery was designed, simulated, and evaluated using BioSTEAM,^32,52^ and the thermodynamic package utilized was Thermosteam.^53,54^ Briefly, influent and effluent streams of each unit are simulated in BioSTEAM and coupled with operating parameters and equipment cost algorithms for unit design and cost calculations. Further descriptions of major processes and units (Section S1 and Table S2) as well as baseline values and uncertainty distributions of key parameters (Table S6) are included in the SI. All Python scripts for BioSTEAM and the biorefinery (including biorefinery setup and system analyses) as well as a system report (including detailed process flowsheet, stream composition and cost tables, unit design specifications, and utilities for the baseline simulation) are available in the online repository.^44^

### System Analyses under Uncertainty

#### Techno-Economic Analysis (TEA) and Life Cycle Assessment (LCA)

We performed TEA and LCA following established procedures for bioproducts and biofuels.^29,49–51,55–57^ Briefly, TEA was executed using BioSTEAM’s discounted cash flow rate of return analysis to calculate the minimum product selling price (MPSP, $·kg^–1^) of TAL to achieve a net present value of zero with a targeted annual internal rate of return.^41–43^ All costs and prices shown are presented in 2019 U.S. dollars. For the baseline case, the targeted annual internal rate of return was 10%, project duration was 30 years, sugarcane unit price was $34.50 per wet metric ton, natural gas unit price was $27.65·kg^–1^, (baseline values and distributions for all parameters included in the uncertainty analysis are detailed in Tables S6 and S7 in the SI with references). Key construction (e.g., warehouse, site development), fixed operating (e.g., labor burden, property insurance), and financial (e.g., depreciation, taxes) parameters followed assumptions in previous studies.^58,59^ We performed LCA in Python using the simulated inventories for streams (input chemicals and output emissions) and utilities from BioSTEAM. The LCA scope included the operational phase of the biorefinery, including cradle-to-grave impacts for all raw materials, ancillary processes, and unit processes.

The baseline impacts of sugarcane farming (excluding credit for fixed carbon), harvest and collection, transportation, storage, and handling were considered. Note that although direct land use change was included in the environmental impacts associated with sugarcane cultivation,^60^ the potential impacts of indirect land use change were not included and continue to be a topic of robust discussion.^61^ The functional unit was set to 1 kg of produced TAL to be consistent with the TEA. The sale of coproduced electricity was assumed to displace the impacts of average grid electricity production (assumed to be equal to the environmental impacts reported in GREET 2022 for the U.S. grid mix^62^). Although uncertainties in the electricity unit impacts may substantially influence the system impacts–including changes in electricity production mixes in response to long-term electricity production by biorefineries–these were not included in this analysis to maintain the focus on biorefinery processes in the uncertainty and sensitivity analyses. Final characterization and discussion of environmental impacts focused on two impact categories selected based on their prominence in the literature and their relevance to policies and legislation: cradle-to-grave carbon intensity (CI; quantified as 100-year global warming potential, GWP_100_) and fossil energy consumption (FEC; quantified as cumulative fossil energy demand).^10,63^ While the mass and energy balances from the simulations performed in this work could be used to assess other impact categories (e.g., acidification, ecotoxicity, global warming potential, carcinogenics, and respiratory effects^64^), the focus of this work was on reducing CI and reliance on fossil energy. Although TAL can be upgraded to other chemicals (e.g., sorbic acid, polydiketoenamines) for a variety of uses, end-of-life emissions assumed all bioderived material would ultimately degrade through passive oxidation to CO_2_. This assumption avoided carbon sequestration in commercial products, and was consistent with previously published LCAs on bioproducts that are upgraded to other chemicals prior to use.^49–51,63^ The geographic boundary of the LCA was the United States, with “rest-of-the-world” inventories used where US inventories were not available (sources for unit inventory values for all raw materials and utilities were noted in the script^44^). A full list of baseline values and distributions for parameters included in the uncertainty analysis (e.g., TEA parameters including raw material prices, biorefinery annual operating time, TAL production capacity, federal corporate tax rate, and targeted internal rate of return) is included in Tables S6 and S7 in the SI. Further details of the TEA and LCA are discussed in Section S1.5 of the SI. A breakdown of the estimated revenue, capital and operating expenditures, CI, and FEC, as well as additional details on the design, utility requirements, purchase costs, and installed equipment costs can be found online.^44^

#### Uncertainty and Sensitivity Analyses

Uncertainty analysis was conducted for the baseline biorefinery design using Monte Carlo simulation with Latin Hypercube Sampling (6000 simulations) for 30 uncertain parameters (Table S6). A detailed description on the choice of parameters distribution type and range is included in Section S1.6 of the SI. By employing a quantitative sustainable design framework,^65^ we performed a global sensitivity analysis as well as targeted local sensitivity analyses to generate insight into the system sustainability implications of potential process-level improvements and other biorefinery decisions. Specifically, the sensitivity of MPSP, CI, and FEC to all uncertain inputs was determined via Spearman’s rank order correlation coefficients (Spearman’s ρ), and parameters to which the sustainability indicators (MPSP, CI, FEC) were most sensitive (i.e., |Spearman’s ρ| ≥ 0.10 and *p*-value < 0.05) were identified for additional analyses. In addition, the improvements in sustainability indicators in response to technological advancements in fermentation and separation were also characterized. For fermentation, indicator sensitivity to fermentation titer (i.e., the final TAL concentration in the fermentation reactor in g·L^–1^), overall yield (i.e., the mass of TAL produced per unit mass of sugars and acetate consumed; the theoretical maximum or 100% of theoretical yields are approximately 0.467 g-TAL·g-glucose eq^–1^ and 0.467 g-TAL·g-acetic acid eq^–1^), and productivity (the mean rate of TAL production in g·L^–1^·h^–1^) were quantified. For separation, indicator sensitivity to TAL ring-opening decarboxylation (mol %; 20.9% in the baseline case) and pH maintained (by addition of sodium hydroxide; pH was 2.10 in the baseline case due to the presence of acids—namely, phosphoric acid added during feedstock pretreatment and citric acid produced during fermentation) were quantified. Finally, we quantified indicator sensitivities to biorefinery annual operating time (days) and TAL production capacity (metric ton·y^–1^). Files with comprehensive results of all analyses are available online.^44^

## Results and Discussion

### Temperature-Sensitive Solubility of TAL in Water

By experimentally measuring TAL solubility in water at various temperatures, we identified TAL solubility in water was highly sensitive to temperature, with a minimum observed value of 3.52 g-TAL·L^–1^ at 0 °C and a maximum observed value of 130.65 g-TAL·L^–1^ at 93 °C (Table S4 in the SI). This disparity demonstrated the potential for a separation process design that exploits the temperature-sensitivity of TAL solubility. We fit the TAL solubility model using one empirical parameter (eq S3 in the SI) to the 12 experimental data points for TAL solubility obtained in this work resulting in a coefficient of determination, *R* ^2^, of 0.992 (Figure 3). While we calibrated other models to experimental solubility data (Figure S3), the model described by eq S3 in the SI was associated with the highest goodness of fit and was therefore used in all models developed in this work (solubility models described in detail in Section S1.3 of the SI).

**Figure 3:**
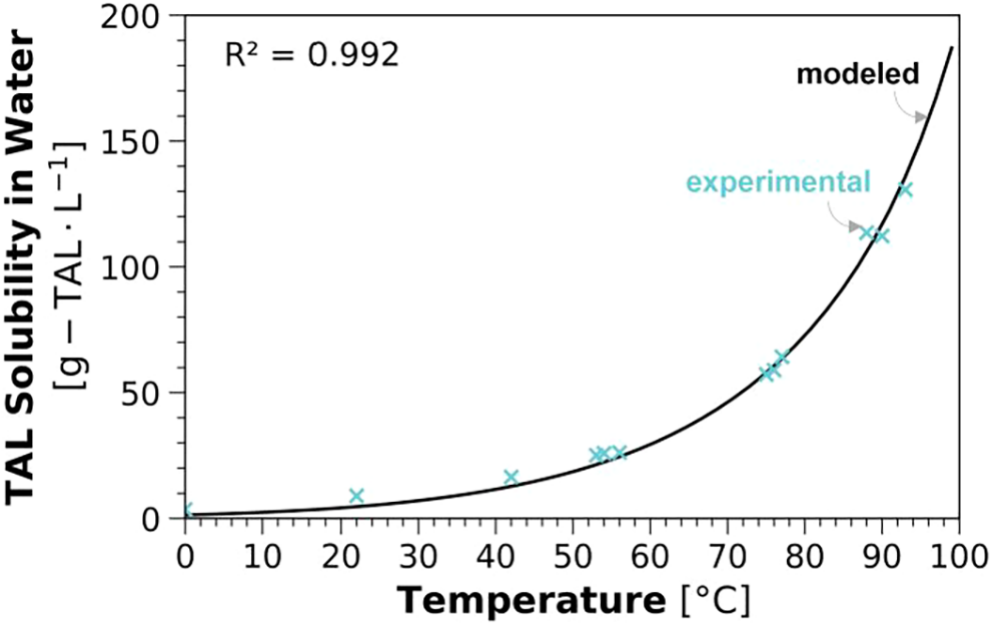
Solubility of TAL in water (g-TAL·L^–1^; *y*-axis) as a function of temperature (°C; *x*-axis). The solubility model with activity coefficients estimated by the one-parameter van Laar method (applying the parameter reduction method suggested by Poling, Prausnitz, and O’Connell^47^ to the equation originally proposed by Wohl^66^) as shown in eq S3 in the SI is plotted (solid black line) along with experimentally observed solubilities used to fit the model (blue cross markers). Methods for solubility measurements and modeling are described in detail in Section S1.3 of the SI.

### Financial Viability under Uncertainty

The MPSP of TAL was estimated to be $4.60·kg^–1^ (baseline) with a range of $3.73–5.86·kg^–1^ [5th–95th percentiles, hereafter shown in brackets]. Overall, for the current state-of-technology under uncertainty and considering TAL to be a feedstock for sorbic acid production, the MPSP achieved by the biorefinery was below the low end of the maximum viable price range ($5.99·kg^–1^) in 96.5% of simulations and below the high end of the maximum viable price range ($7.74·kg^–1^) in 100.0% of the simulations, indicating the designed biorefinery can be financially viable (Figure 4A). Further, considering TAL to be a dimedone replacement as a feedstock to produce polydiketoenamine plastics, the TAL MPSP was below the benchmark price of $10·kg^–1^ in 100.0% of simulations, indicating a high likelihood of financial viability for this alternative product (Figure 4A). Process contributions to the biorefinery’s total capital cost, annual operating cost, and utility use for heating, cooling, and power demands are broken down under uncertainty and discussed in detail in Section S2.1 of the SI.

**Figure 4:**
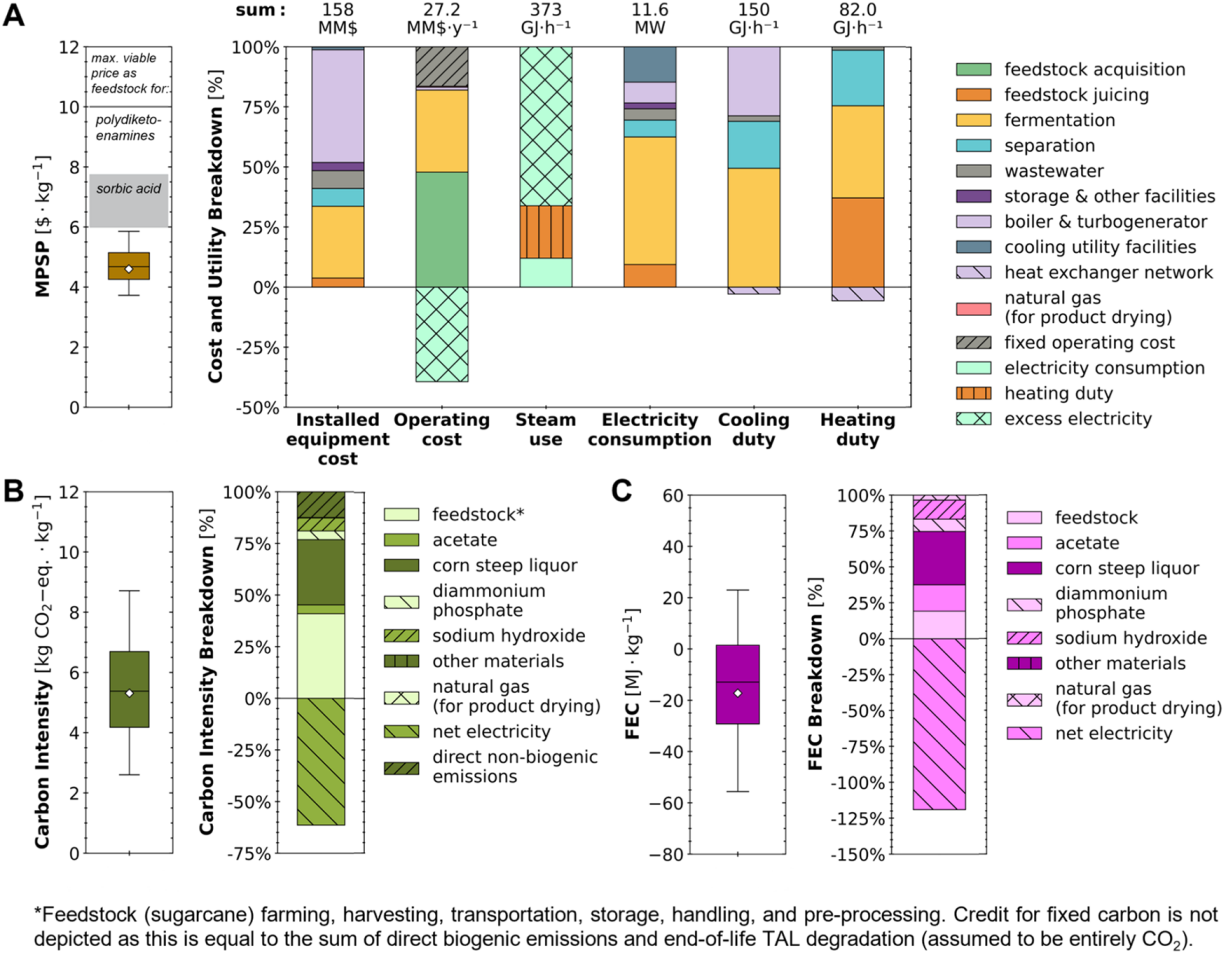
Uncertainties (box-and-whisker plots) and breakdowns (stacked bar charts) for (A) minimum product selling price (MPSP), (B) carbon intensity (CI) quantified as 100-year global warming potential (GWP_100_), and (C) fossil energy consumption (FEC) per kg of TAL produced via fermentation of glucose and acetate by *Y. lipolytica*. On box-and-whisker plots, whiskers, boxes, and the middle line represent 5th/95th, 25th/75th, and 50th percentiles, respectively, from 6000 Monte Carlo simulations. Diamonds and stacked bar charts report results for baseline values. The shaded gray regions show the maximum viable price range for TAL as a dimedone replacement to produce polydiketoenamine plastics ($10·kg^–1^; the market price for dimedone)^10^ and as a feedstock for sorbic acid ($5.99–7.74·kg^–1^; based on the market price range for sorbic acid of $6.74–8.71·kg^–1^).^11,37^ For the biorefinery’s operating cost (MM$·y^–1^), contributions from fixed operating costs, sales revenue from excess electricity, purchase of natural gas for product drying, and material costs by process area are shown. Values above stacked bars are totals including offsets. For heating duty, cooling duty, and electricity consumption, values indicate totals during operation. The biorefinery is assumed to operate 180 days annually at the baseline condition (baseline values and distributions with literature references for all parameters are detailed in Table S6 in the SI). TAL carbon intensity included end-of-life emissions (2.09 kg CO_2_-eq·kg^–1^) assumed to be entirely through the passive oxidation of TAL to CO_2_. Tabulated data breaking down capital and material costs, heating and cooling duties, electricity consumption, CI, and FEC are available online.^44^

Leveraging the Monte Carlo simulations, the sensitivity of MPSP to the 30 uncertainty parameters was characterized via Spearman’s rank order correlation coefficients. The full sensitivity analysis results are presented in Section S2.2 and Figure S8 in the SI. Briefly, these results indicate the fermentation process, separation process, TAL production capacity, and operating schedules may offer significant opportunities for improvements to achieve financially viable TAL production. Accordingly, the implications of potential improvements to fermentation and separation and the implications of alternative production capacities and operating schedules are explored and discussed in the subsequent sections.

### Environmental Impacts under Uncertainty

The baseline cradle-to-grave CI and FEC impacts of TAL production were estimated to be 5.31 [2.60–8.71] kg CO_2_-eq·kg^–1^ and −17.2 [−55.6–22.9] MJ·kg^–1^, respectively (Figure 4B,C), with net displacement of fossil energy consumption (i.e., FEC < 0) in 72.7% of simulations. The biorefinery’s CI was lower than the benchmark dimedone CI (8.0 kg CO_2_-eq·kg^–1^)^10^ in 89.9% of simulations. The CI was substantially lower than that estimated by a previous LCA^10^ (approximately 14 kg CO_2_-eq·kg^–1^), which may be explained by the substantially improved fermentation performance assumed in this study (TAL yield of 0.19 g·g^–1^ rather than 0.09 g·g^–1^ substrates; TAL titer of 35.9 rather than 2.8 g·L^–1^). Coproduced electricity was assumed to displace impacts from the production of marginal grid electricity (Figure 4B,C) as recommended in the U.S. Renewable Fuel Standard (RFS^67^). The total CI and FEC were the sum of the total positive impacts (13.76 [10.22–17.64] kg CO_2_-eq·kg^–1^ and 90.5 [66.5–117.1] MJ·kg^–1^, respectively) and the offsets from coproduced electricity (−8.45 [−11.93 to −4.98] kg CO_2_-eq·kg^–1^ and −107.8 [−152.1 to −63.5] MJ·kg^–1^, respectively). Process contributions to the biorefinery’s CI and FEC are broken down under uncertainty and discussed in detail in Section S2.1 of the SI.

Consistent with MPSP, sensitivity analysis results (Section S2.2 and Figure S8 in the SI) indicate the fermentation (including TAL titer and yield) and separation (including TAL ring-opening decarboxylation) processes may offer significant opportunities to mitigate the biorefinery’s environmental impacts. Accordingly, the implications of potential fermentation and separation improvements are quantified and discussed in the subsequent sections.

### Prioritization of Technology Development and Scale-Up Pathways

As the sensitivity analysis highlighted (Section S2.2 and Figure S8 in the SI), the performance of the fermentation unit has significant implications for MPSP, CI, and FEC. This is consistent with the conclusions of previous works that have highlighted the need for identifying and pursuing specific targets for fermentation parameters in biological TAL production.^5,10,30^ To this end, we designed and simulated the biorefinery across the entire titer-yield theoretical performance space (i.e., 3600 potential yield-titer combinations) for a range of productivities to quantify how future improvements to microbial conversion (e.g., via synthetic biology) would impact the sustainability of sugar-based TAL production.

Across the evaluated theoretical fermentation space, MPSP benefited from increased yield and titer of TAL, with a potential minimum of $2.11·kg^–1^ as yield approached 99% theoretical and titer approached 100 g·L^–1^ (Figure 5A). The relative impact of fermentation yield vs titer improvements depended on the location in the yield-titer performance space. In general, improvements to yield were more impactful at higher titer values and improvements to titer were more impactful at high-yield points. Improvements to yield would increase the biorefinery’s total capital cost and operating cost (Figure S10A in the SI) but increase TAL production enough to result in an overall reduction in MPSP. At a fixed productivity, improvements to titer would lead to a longer fermentation time and thus a more expensive conversion process; however, titer improvements would result in an overall reduction to the MPSP by reducing the biorefinery’s total capital cost (Figure S10A) as less dilute streams require smaller equipment sizes, and by reducing the annual operating cost as a higher titer enables less utility-intensive separations (Figure S10B).

**Figure 5:**
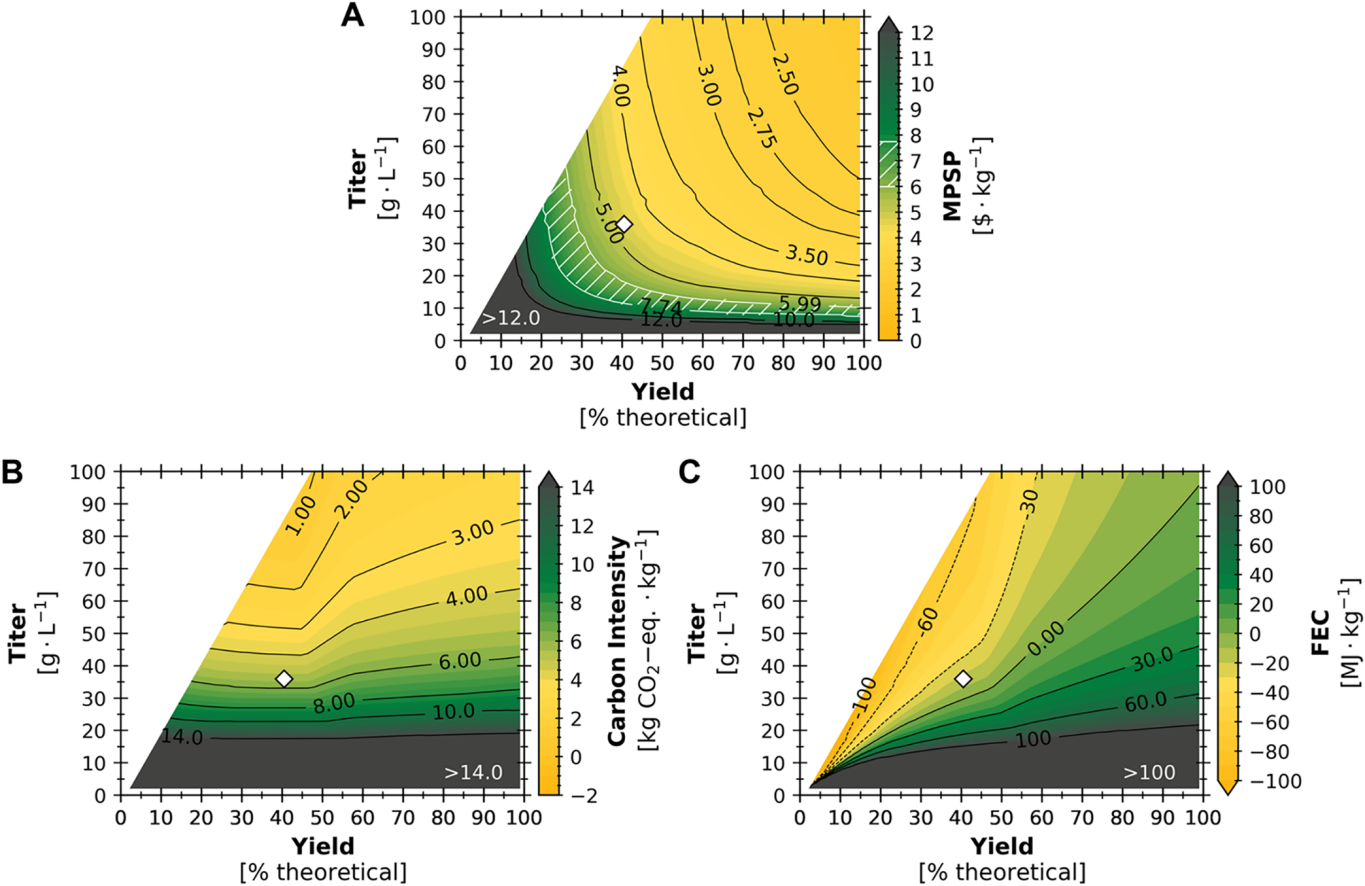
(A) Minimum product selling price (MPSP), (B) life cycle carbon intensity (CI), and (C) fossil energy consumption (FEC) of the produced TAL across theoretical fermentation TAL yields (*x*-axes) and titers (*y*-axes) at baseline productivity (0.12 g·L^–1^·h^–1^). For a given point on the figure, the *x*-axis value represents the overall fermentation TAL yield (as the percent of maximum theoretical yield of TAL on glucose, sucrose, and acetate, where the maximum theoretical yield is assumed to be 0.467 g·g-glucose-eq^–1^ and 0.467 g·g-acetic-acid-eq^–1^), the *y*-axis value represents the titer, and the color represents MPSP, CI, or FEC. The white region to the upper left of each plot represents infeasible yield-titer combinations (described in Section S1.1 of the SI). The maximum viable TAL price range as a feedstock for sorbic acid production is represented (in A) by hatching with white diagonal lines between $5.99–7.74·kg^–1^. The benchmark of $10·kg^–1^ as a feedstock replacing dimedone for polydiketoenamine plastics production is represented (in A) as a standard contour line. The baseline yield-titer combination (represented by diamonds) constitutes a yield of 40.5% theoretical and a titer of 35.9 g·L^–1^.

At the baseline–fermentation yield of 40.5% theoretical and titer of 35.9 g·L^–1^ (diamond marker in Figure 5A)–incremental improvements in yield would have a greater benefit to MPSP than incremental improvements in titer. For instance, a 10% relative improvement over the baseline to fermentation yield (to 44.5% of theoretical) would reduce the MPSP by $0.32·kg^–1^ TAL, while a 10% relative improvement to fermentation titer (to 39.5 g·L^–1^) would reduce the MPSP by only $0.14·kg^–1^. However, at higher values for fermentation yield (e.g., 88.0% of theoretical), a 10% relative improvement to yield (to 96.8% of theoretical) would only reduce MPSP by $0.11·kg^–1^, while a 10% relative improvement to titer (to 39.5 g·L^–1^) would reduce the MPSP by a comparable $0.10·kg^–1^. Ultimately, improvements to yield alone (relative to the baseline) could only reduce MPSP to a potential minimum of $2.83·kg^–1^, and improvements to both titer and yield would be needed to achieve the potential minimum of $2.11·kg^–1^ in the evaluated theoretical fermentation space (Figure 5A).

While baseline MPSP benefited from incremental improvements to both fermentation yield and titer, fermentation titer presents much greater opportunities to benefit CI and FEC. This finding is illustrated by the slope of the contour lines for CI and FEC near the baseline (diamond markers in Figure 5B,C). This observation stems from the fact that although baseline feedstock and acetate acquisition together accounted for around 45% and 38% of detrimental contributions to CI and FEC, respectively, the baseline excess electricity production resulted in offsets of 61% and 119% to CI and FEC, respectively (Figure 4B,C). If fermentation TAL yield increases, feedstock and acetate acquisition contributions to CI and FEC would be reduced, but the production of cell mass and citrate (both of which had negative Spearman’s ρ values for CI and FEC; Figure S8 in the SI) would also decrease. The result of this shift would be a reduction in the energetic content of waste streams diverted to anaerobic treatment (in wastewater management) to produce biogas for combustion in the boiler, which regenerates steam utilities used for electricity production by the turbogenerator. For instance, if fermentation TAL yield were increased from 40.5% to 70% of theoretical yield at a constant titer of 35.9 g·L^–1^, CI would increase from 5.31 kg CO_2_-eq·kg^–1^ to 6.64 kg CO_2_-eq·kg^–1^ and FEC would increase from −17.2 MJ·kg^–1^ to 28.2 MJ·kg^–1^. However, higher fermentation TAL yields (than the baseline) are required to unlock higher TAL titers, and a minimum CI of 0.18 kg CO_2_-eq·kg^–1^ is potentially achievable with improvements to both titer and yield (Figure 5B). For FEC, low-yield, high-titer combinations resulted in the lowest FEC values (<−100 MJ·kg^–1^) as this resulted in high-energy waste streams available for biogas production in the anaerobic digester, enabling higher production of excess electricity. However, if electricity offsets were not considered, the CI and FEC would improve monotonically with both fermentation TAL yield and titer (Figure S11 in the SI).

Further, we found increasing productivity to 500% of the baseline (i.e., to 0.60 g·L^–1^·h^–1^) would not substantially change CI or FEC and only decrease MPSP by 13% (Section S2.3 and Figure S5 in the SI). Although major productivity improvements could substantially reduce the biorefinery’s total capital cost (e.g., an 18% decrease when productivity is increased to 500% of the baseline; Section S2.3 of the SI), and although major changes in fermentation productivity (relative to the baseline) may significantly impact MPSP (e.g., a 61% increase when productivity is decreased to 20% of the baseline; Figure S4 in the SI), improvements to titer and yield offer the most significant opportunities to further enhance the financial viability and environmental sustainability of biobased TAL production.

Given the sustainability indicators (MPSP, CI, and FEC) were sensitive to fermentation TAL yield and titer (Figure S8 in the SI), the following targeted improvements were explored to illustrate the potential benefits of additional microbial conversion research and development: (i) fermentation TAL yield increase from 40.5% (0.19 g·g^–1^) to 73.0% of theoretical (0.34 g·g^–1^, comparable to the reported yield of 0.39 g·g^–1^ using *E. coli* to produce adipic acid,^68^ another 6-carbon metabolite with low solubility in water); and (ii) fermentation TAL titer increase from 35.9 g·L^–1^ to 68.0 g·L^–1^ (equal to the reported adipic acid titer of 68.0 g·L^–1^ achieved using *E. coli* ^69^). If these two targets are achieved, the resulting MPSP of TAL ($3.14·kg^–1^ [$3.03–4.37·kg^–1^]) would be lower than the maximum viable price range as a sorbic acid feedstock ($5.99–7.74·kg^–1^) in 100.0% of simulations, and the resulting CI (3.35 [1.93–4.82] kg CO_2_-eq·kg^–1^) would be lower than the benchmark dimedone CI (8.0 kg CO_2_-eq·kg^–1^) in 100.0% of simulations (Figure S9A in the SI; the implications of these targeted fermentation improvements on MPSP, CI, and FEC are further discussed in Section S2.3 of the SI).

Beyond fermentation improvements, the sensitivity analysis performed for the baseline scenario highlighted the significance of operating time and TAL production capacity on the economics of the biorefinery (Section S2.2 and Figure S8 in the SI). While the baseline TAL production capacity was 13385 metric tons TAL·y^–1^, there is significant potential for larger production capacities to meet current and projected U.S. and global demands for a range of potential products for which TAL can serve as a feedstock (including sorbic acid,^2,6^ polydiketoenamine plastics,^10^ acetylacetone,^2^ pogostone,^7^ katsumadain,^8^ and penicipyrone,^9^ among others). Further, there is large uncertainty in the operating schedule for sugarcane biorefineries (e.g., 120–200 annual operating days^41–43^ ), and additionally accepting sweet sorghum as a feedstock (as the composition is similar to sugarcane^41^) could significantly increase biorefinery operating time (e.g., to 240 annual operating days^41^). To quantify the economic implications of alternative biorefinery operating times and TAL production capacities, we simulated and evaluated the biorefinery across the production-operation space (i.e., 6400 potential combinations of biorefinery operating time and TAL production capacities; Figure S6, discussed in detail in Section S2.4 of the SI).

Finally, the sensitivity analysis highlighted the significance of TAL loss by ring-opening decarboxylation (Section S2.2 and Figure S8 in the SI), which is reportedly initiated by the reversible keto–enol tautomerization of TAL, followed by nucleophilic addition of water to the lactone carbonyl, both steps that require the presence of protons (H^+^) in solution.^46^ Therefore, we simulated the biorefinery across potential improvements to TAL recovery in the separation process by controlling pH through base addition (i.e., 3600 potential combinations of maintained pH and TAL loss by ring-opening decarboxylation). We found if a pH of 11.0 maintained by sodium hydroxide addition were sufficient to decrease TAL ring-opening decarboxylation conversion during separation from 20.9 mol % (baseline) to 4.8 mol %, the MPSP would be reduced to $4.35·kg^–1^ ($0.51·kg^–1^ lower than the baseline), and the CI would increase slightly (by 0.57 kg CO_2_-eq·kg^–1^) to 4.37 kg CO_2_-eq·kg^–1^ (Figure S7, discussed in detail in Section S2.5 of the SI).

If the discussed potential improvements to fermentation (increasing yield to 73.0% theoretical and titer to 68.0 g·L^–1^) were achieved in combination with the integrated processing of sweet sorghum during two months (May and September) when sugarcane is not harvested in the southern U.S.^41^ (increasing annual operating time to 240 days and TAL production capacity to 17869 metric tons TAL·y^–1^) and potential improvements to separation (decreasing TAL ring-opening decarboxylation conversion to 4.8 mol % by maintaining a pH of 11.0; baseline values and uncertainty distributions for all parameters included in uncertainty analyses are detailed in Tables S6 and S7 in the SI), the resulting biorefinery could produce TAL at an MPSP of $2.26·kg^–1^ [$1.97–2.80·kg^–1^] with a CI of 3.05 [1.91–4.15] kg CO_2_-eq·kg^–1^ and FEC of 3.0 [−13.5–17.3] MJ·kg^–1^. In 100.0% of simulations, the biorefinery’s MPSP was lower by at least $2.42·kg^–1^ and $6.43·kg^–1^ than the maximum viable price range for TAL as a feedstock for sorbic acid ($5.99–7.74·kg^–1^) and as a replacement for dimedone as a polydiketoenamine feedstock ($10·kg^–1^), respectively (Figure S9A in the SI). Further, the biorefinery’s CI was lower by at least 2.81 kg CO_2_-eq·kg^–1^ than the benchmark dimedone CI (8.0 kg CO_2_-eq·kg^–1^) in 100.0% of simulations (Figure S9B,C in the SI), highlighting the potential for combined improvements in fermentation, separation, and feedstock integration to further enhance the biorefinery’s financial viability and environmental benefits.

### Conclusions and Path Forward

In this study, we leveraged BioSTEAM in Python to automate the design, simulation, TEA, and LCA for production of TAL from sugarcane. Under the current state-of-technology (i.e., baseline performance), the MPSP of the produced TAL was $4.60·kg^–1^ [$3.73–5.86·kg^–1^], which was below the low end of the maximum viable price range as a sorbic acid feedstock ($5.99·kg^–1^) in 96.5% of simulations, below the high end of the maximum viable price range as a sorbic acid feedstock ($7.74·kg^–1^) in 100.0% of the simulations, and below the benchmark price to replace dimedone as a feedstock for polydiketoenamine plastics ($10·kg^–1^) in 100.0% of simulations in the uncertainty analysis. This indicates the designed biorefinery may be financially viable at the current state-of-technology under uncertainty with *n*th plant assumptions. The carbon intensity (CI of 5.31 [2.60–8.71] kg CO_2_-eq·kg^–1^) and FEC (−17.2 [−55.6–22.9] MJ·kg^–1^) benefited significantly from the coproduction of excess electricity, which was assumed to displace the environmental impacts of marginal grid electricity production, with net displacement of fossil energy consumption in 72.7% of simulations. Improvements in key technological parameters (especially related to fermentation), design strategies (e.g., to mitigate TAL ring-opening decarboxylation during separation by pH control), and sweet sorghum integration (to increase operating time and TAL production capacity) could significantly reduce the environmental impacts and further improve financial viability.

Improvements in key technological parameters could substantially reduce the environmental impacts and further improve financial viability. If targeted incremental improvements to fermentation TAL yield (to 73.0% of theoretical) and titer (to 68.0 g·L^–1^) were combined with sweet sorghum integration (increasing biorefinery annual operating time to 240 days and TAL production capacity to 17869 metric tons TAL·y^–1^) and potential improvements to separation (decreasing TAL ring-opening decarboxylation to 4.8 mol % by maintaining a pH of 11.0), the resulting biorefinery’s financial viability would be further enhanced (MPSP of $2.26·kg^–1^ [$1.97–2.80·kg^–1^] with a further reduced CI of 3.05 [1.91–4.15] kg CO_2_-eq·kg^–1^ and FEC of 3.0 [−13.5–17.3] MJ·kg^–1^). The uncertainties in CI and FEC would be significantly mitigated through robust characterization of nutrient requirements (specifically, nitrogen and phosphorus), which directly influence chemical inputs to support the microbial conversion.

Other opportunities to advance system sustainability include improving microbial co-utilization of glucose and xylose to enable the use of lignocellulosic feedstocks (e.g., corn stover, miscanthus grass, switchgrass, which have the potential for greater environmental benefits relative to first-generation feedstocks) and designing strategically integrated facilities that accept a mix of renewable feedstocks to produce portfolios of bioproducts and bioenergy optimized to local contexts. System financial viability would be further advanced with government incentives and support for TAL production from renewable feedstocks such as sugarcane, sweet sorghum, and lignocellulosic biomass. Overall, the conclusions from this study support the continued development of TAL production from renewable feedstocks and illustrate how agile and robust system analyses can elucidate key drivers of system cost and environmental impacts, examine the entire feasible technology space, navigate economic and environmental trade-offs, screen promising designs, avoid false precision, and prioritize future research, development, and deployment pathways.

## Supplementary Material





## Data Availability

All results (including plots and raw data) and the software scripts used to generate the same are available at *BioSTEAMDevelopmentGroup: Triacetic acid lactone biorefineries, 2025* (https://github.com/BioSTEAMDevelopmentGroup/Bioindustrial-Park/tree/master/biorefineries/TAL).

## Notes

Supplementary process description and analysis methods (Section S1); supplementary results (Section S2); simplified overview of the designed biorefinery, system sustainability across simulated scenarios , modeled TAL solubility, sensitivity analysis results (Section S3), and assumed composition of feed sugarcane , list of major units and equipment included in the biorefinery, and additional supporting data and assumptions (Section 4) (PDF)
Baseline system report (XLSX)
