# Supplementary material for: Sustainable Triacetic Acid Lactone Production from Sugarcane by Fermentation and Crystallization
Source: ACS Sustain Chem Eng. 2025 Oct 16;13(42):17794–805. doi: 10.1021/acssuschemeng.5c04797 (PMC12570263; doi:10.1021/acssuschemeng.5c04797)
Supplement: Supplementary file 1 [file sc5c04797_si_001.pdf]

Supporting Information (SI) for

Sustainable Triacetic Acid Lactone Production from Sugarcane by Fermentation and Crystallization

Sarang S. Bhagwat[1,2], Marco N. Dell'Anna[1,3], Yalin Li[1,4], Mingfeng Cao[1,5], Emma C. Brace[1,6], Sunil S. Bhagwat[7,8], George W. Huber[1,3], Huimin Zhao[1,5], Jeremy S. Guest[1,2,9,*]

[1]DOE Center for Advanced Bioenergy and Bioproducts Innovation (CABBI), University of Illinois Urbana-Champaign, 1206 W. Gregory Drive, Urbana, IL 61801, USA

[2]The Grainger College of Engineering, Department of Civil and Environmental Engineering, University of Illinois Urbana-Champaign, 3221 Newmark Civil Engineering Laboratory, 205 N. Mathews Avenue, Urbana, IL 61801, USA

[3]Department of Chemical and Biological Engineering, University of Wisconsin-Madison, 1415 Engineering Drive, Madison, WI 53706, USA

[4]Department of Civil and Environmental Engineering, Rutgers, The State University of New Jersey, 500 Bartholomew Rd, Piscataway, NJ 08854, USA

[5]Department of Chemical and Biomolecular Engineering, University of Illinois Urbana-Champaign, 215 Roger Adams Laboratory, 600 S. Mathews Avenue, Urbana, IL 61801, USA

[6]Department of Engineering, Boston College, 245 Beacon St, Chestnut Hill, MA 02467, USA

[7]Department of Chemical Engineering, Institute of Chemical Technology, Nathalal Parekh Marg, Matunga, Mumbai, Maharashtra 400019, India

[8]Department of Chemistry, Indian Institute of Science Education and Research (IISER) Pune, Dr. Homi Bhabha Road, Pune, Maharashtra 411008, India

[9]Institute for Sustainability, Energy, and Environment (iSEE), University of Illinois Urbana-Champaign, 1101 W. Peabody Drive, Urbana, IL 61801, USA

*Corresponding author; email: jsguest@illinois.edu

Number of pages: 41

Number of figures: 12

Number of tables: 9

Table of Contents

Section S1. Supplementary Process Description and Analysis Methods 3

S1.1. Fermentation Process 3

S1.2. Experimental Measurement of TAL Solubility and Ring-Opening Decarboxylation in Water 4

S1.3. Solubility Models Calibrated 5

S1.4. Estimating the pH of Simulated Streams 6

S1.5. Details of Biorefinery Techno-Economic Analysis and Life Cycle Assessment 7

S1.6. Selection of Uncertainty Distributions 8

S1.7. Facilities 9

Section S2. Supplementary Results 10

S2.1. Breaking Down Process Contributions to System Costs and Environmental Impacts ...10

S2.2. Global Sensitivity Analysis Results 12

S2.3. System Sustainability Implications of Targeted Fermentation Improvements 12

S2.4. Market-Driven Capacity Expansion and Operating Schedule Considerations 13

S2.5. Exploring Potential Separation Improvements by pH Control 14

Section S3. Supplementary Figures 16

Section S4. Supplementary Tables 26

References 37

Section S1. Supplementary Process Description and Analysis Methods

S1.1. Fermentation Process

The fermentation mode was assumed to be fed-batch. The retention time for the fermentation reactor was determined through the designated titer and productivity.

The clarified sugarcane juice stream needs further processing so that the sucrose, glucose, and fructose are present at the concentrations required to achieve the targeted TAL concentration. To facilitate this, the saccharified stream is either passed through a multiple-effect evaporator which concentrates the stream (to no more than 600 g·L^{-1} of total sugars to avoid deposition of solids) or diluted as needed, such that the final concentration of TAL in the fermentation reactor is the target concentration (hereafter referred to as the titer). For the baseline design, a vapor fraction of zero for the evaporator paired with a dilution increase of 0.124 kg water per kg original clarified juice (i.e., increasing the water content of the clarified juice from 81.72 wt% to 83.74 wt%) was utilized to achieve the baseline TAL titer of 35.9 g·L^{-1} after fermentation.

As the multiple effect evaporator is set to concentrate the clarified juice to no more than 600 g·L^{-1} of total sugars, there is an implicit maximum TAL titer that can be achieved at any given yield on sugars during fermentation. This manifests as an infeasible region in the upper-left portion of the fermentation titer-yield space (represented by white regions in **Figures 4**, **S4**, **S5**, **S9**, and **S10**). Experimental fermentation metrics (e.g., titer, yield, productivity) were used to guide the design of the conversion process (**Table S3**), which were collected from literature on microbes converting sugars and/or acetate into TAL. The collected literature showed us that *Yarrowia lipolytica* is currently the most promising six-carbon sugar-utilizing microbial candidate with the highest TAL titer and yield. To the best of our knowledge, only 5 existing papers have analyzed the fermentation performance of any species of *Y. lipolytica* on sugars and/or acetate to produce TAL (**Table S3**). Of those 5 papers, one explored co-utilization of glucose and xylose in corn stover hydrolysate and achieved an impressive titer of 21.4 g·L^{-1}; however, we were not able to estimate their achieved yield from the reported data (**Table S3**). Of the remaining 4 papers, Markham et al. developed a strain of *Y. lipolytica* capable of producing TAL with a titer of 35.9 g·L^{-1} and a yield of 40.5% of the theoretical maximum on glucose and acetate via fed-batch fermentation,[1] the highest TAL yield and titer reported in the literature (**Table S3**). The highest titers reported by fermenting only sugars without the use of acetate were 2.6 g·L^{-1} by *Y. lipolytica* using glucose[2,3] and 2.9 g·L^{-1} by *Y. lipolytica* using xylose[2] (**Table S3**). Thus, the *Y. lipolytica* strain developed by Markham et al.[1] was selected as the current representative of baseline performance for fermentation of sugars and/or acetate to produce TAL. The North American market size for acetic acid in 2022 was 1.58 million metric tons,[4] and the projected 10-year growth (an additional 874,000 metric tons by 2032[4]) is far greater than required to accommodate the 4,882 metric tons of sodium acetate (in acetic acid mass equivalents, assuming 100% conversion of acetic acid to sodium acetate by neutralization) required annually for the baseline biorefinery. Note although the use of sodium acetate could introduce sodium ions in the TAL product, the implications of sodium presence on catalytic upgrading (e.g., to potassium sorbate) were not explored.

In the bioreactor-scale experiments performed by Markham et al.[1] for conversion of sugars to TAL by *Y. lipolytica*, the media was reported to contain 40 g·L^{-1} peptone and 20 g·L^{-1} yeast extract to provide nitrogen for microbial growth. However, these nitrogen sources are generally expensive, and corn steep liquor (CSL) has been suggested as a potential, more economically feasible alternative for large-scale fermentation processes.[5] Therefore, we assumed an equivalent amount of CSL (in terms of total nitrogen) to be used instead of yeast extract and peptone as a nitrogen source. To characterize the implications of the uncertainty in the media composition required, we considered the total nitrogen contents of yeast extract (10.0–11.8 dry wt%), peptone (13.717 dry wt% by conservatively assuming 100% of the peptone is tryptophan,

which is a major constituent[6]), CSL (7.7–8.2 dry wt%), and the *Y. lipolytica* cell mass (roughly 0.07155 g N·g cells^{-1} from Niehus et al.[7]). As we could not easily determine the final amount of cell mass (including viable and non-viable cell mass) from the viable cell count reported by Markham et al., we instead assumed a final amount of cell mass of 47.8 g·L^{-1} (corresponding to a cell mass yield of 0.146 g-cells·g-glucose-eq^{-1}), which was the amount reported by Cordova et al.[8] for *Y. lipolytica* grown using corn stover hydrolysate with a final TAL titer of 21.6 g·L^{-1}. In addition to nitrogen, we assumed phosphorus would be required for microbial growth, and modeled diammonium phosphate (DAP) as the phosphorus source based on the amount reportedly provided per unit mass of CSL provided for ethanol-producing yeast in a previous study by Humbird et al.[5] (roughly 0.133 g DAP·g CSL^{-1}). These assumptions resulted in lower-bound estimates of 41.71 g CSL·L^{-1} and 5.55 g DAP·L^{-1} and higher-bound estimates of 112.1 g CSL·L^{-1} and 14.91 g DAP·L^{-1} for CSL and DAP, respectively, and uniform distributions bound by these estimates were assumed for CSL and DAP requirements in the uncertainty analyses performed in this study (**Table S6**).

A dissolved oxygen (DO) saturation of 50% reported by Markham et al.[1] was assumed for the baseline, with an aeration rate safety factor uniformly distributed between 0.5–2 for the uncertainty analyses. To model aeration requirements, we assumed an oxygen solubility in water of 7.8 mg·L^{-1} (based on the reported solubility at 30°C[9]), and calculated the aeration rate required to maintain the targeted DO saturation in the fermentation broth after all reactions were completed, some of which consumed oxygen (e.g., citric acid production, sugar breakdown for energy; all modeled reactions are detailed in the script[10]).

Markham et al.[1] reported controlling the pH during fermentation at 6.5 using sodium hydroxide. We estimated the pH of the simulated broth as well as the concentration of sodium hydroxide required to maintain a pH of 6.5 (method described in **Section S1.4**) and simulated purchase and use of sodium hydroxide to meet these requirements. Sodium hydroxide was simulated to react with citric acid co-produced during fermentation to form sodium citrate, which was assumed to remain dissolved due to its high solubility of approximately 660 g·L^{-1} water even at pH 7.0 and a temperature of 30°C.[11]

A list of all units comprising the biorefinery, including fermentation process units, is included in **Table S1**. A comprehensive analysis on the fermentation unit across the titer-yield performance space at varying productivities was also conducted (**Figures 4**, **S4**, **S5**, **S9**, and **S10**).

S1.2. Experimental Measurement of TAL Solubility and Ring-Opening Decarboxylation in Water

We investigated the effect of varying temperature on TAL solubility in water. An aqueous solution was prepared by adding a specific amount of TAL (Sigma-Aldrich) for a desired solubility concentration. Masses of TAL and water were measured over a precision scale (Mettler Toledo). The solution was then stirred at 400 rpm and slowly heated over a magnetic stirrer hotplate (IKA) with a multi-vial block and a thermocouple to reach the desired temperature. TAL was slowly added until crystals either visibly deposited at the bottom of the vial or floated in solution for a period longer than 5 minutes. This was repeated for each desired temperature (**Table S4**).

In addition, it was observed that heating TAL dissolved in water for extended periods of time resulted in TAL ring-opening decarboxylation to 2,4-pentanedione (acetylacetone). Therefore, we investigated the effect of varying temperature on TAL ring-opening decarboxylation conversion. 10 mL of a solution of 12.2 g·L^{-1} of TAL in water was prepared in a scintillation vial, and poured into a 10-mL conical flask. Three 10-mL conical flasks of the solution (each containing 10 mL of the solution) were prepared in this way. One such conical flask was then placed on a magnetic stirrer hotplate with thermocouple, stirred at 400 rpm, and heated for 1 hour at a temperature of

30°C, while the other two flasks were maintained at 50°C and 80°C. For each flask, after quenching, the final mass of the solution was measured to account for the solvent (water) evaporation. After 1 hour, samples were taken from each flask to measure the conversion of TAL to acetylacetone by gas chromatography analysis (method consistent with that reported by Chia et al.[12]).

S1.3. Solubility Models Calibrated

In a binary system, assuming the solid phase is pure, at temperature T (in K), the solubility x_2 (as a mole fraction; e.g., mol-TAL·mol-solution^{-1}) of a solid solute may be expressed as follows (from Equation 8-16.3 in [13]):

$$\ln \gamma_2 x_2 = \frac{-\Delta H_m}{RT}\left(1 - \frac{T}{T_m}\right) \qquad \text{(Equation S1)}$$

where γ_2 is the activity coefficient of the solute, ΔH_m is the enthalpy change for melting the solute at the triple-point temperature, T_m is the normal melting temperature (in K), and R is the molar gas constant (8.3145 J·mol^{-1}·K^{-1}).

The activity coefficient (γ_2) of the solute may be expressed using a one-parameter van Laar equation as follows (applying the parameter reduction method suggested by Poling, Prausnitz, and O'Connell[13] to the equation originally proposed by Wohl[14]):

$$\ln \gamma_2 = \frac{A}{RT}\left(1 + \frac{V_2^L}{V_1^L}\frac{x_2}{(1-x_2)}\right)^{-2} \qquad \text{(Equation S2)}$$

where V_1^L and V_2^L are the liquid molar volumes of the solvent and solute, respectively, and A is an empirical constant.

From **Equations S1** and **S2**, we obtain:

$$x_2 = e^{\frac{-\Delta H_m}{RT}\left(1-\frac{T}{T_m}\right)-\frac{A}{RT}\left(1+\frac{V_2^L}{V_1^L}\frac{x_2}{(1-x_2)}\right)^{-2}} \qquad \text{(Equation S3)}$$

which is an implicit equation that may be used to numerically solve for x_2 at a given value of T. Although other equations were evaluated and fit to the experimental data (described below), **Equation S3** was exclusively used to estimate TAL solubility for all simulations in this work because of the high goodness of fit obtained for 12 experimental data points using one empirical constant, A.

The enthalpy change of fusion ΔH_m of TAL at the triple-point temperature was estimated to be 30883.7 J·mol^{-1} using the Dannenfelser-Yalkowsky method.[13,15] For the melting temperature T_m of TAL, a reported experimental value of 458.15 K was used.[16] For the liquid molar volume of water V_1^L, a reported experimental value of 1.801 *10^{-5} m^3·mol^{-1} was used.[17] The liquid molar volume of TAL V_2^L was estimated to be 8.85 *10^{-5} m^3·mol^{-1} using the Fedors method.[18]

The empirical constant A was estimated for **Equation S3** by using the experimental values obtained in this work for TAL solubility in water at various temperatures and maximizing the coefficient of determination, R^2. This resulted in a value of 7029.6 J·mol^{-1} for A in **Equation S3**, with R^2 maximized to a value of 0.992 (**Figure 3**).

Alternatively to using the one-parameter van Laar equation as in **Equation S2** to describe the activity coefficient (γ_2) of the solute, γ_2 may be expressed using a one-parameter Margules equation as follows (from Equation 8.16.6 in [13]):

$$\ln \gamma_2 = \frac{B}{RT}(1 - x_2)^2 \qquad \text{(Equation S4)}$$

where B is an empirical constant. S

From **Equations S1** and **S4**, we obtain:

$$x_2 = e^{\frac{-\Delta H_m}{RT}\left(1-\frac{T}{T_m}\right)-\frac{B}{RT}(1-x_2)^2} \quad \text{(Equation S5)}$$

which is an implicit equation that may be used to numerically solve for x_2 at a given value of T.

Note if $x_2 \ll 1$ within the range of values for temperature T across which solubility is desired to be modeled, it can be reasonably assumed that $(1 - x_2)^2 \approx 1$ within that temperature range. This assumption would result in the following equation:

$$x_2 = e^{\frac{-\Delta H_m}{RT}\left(1-\frac{T}{T_m}\right)-\frac{C}{RT}} \quad \text{(Equation S6)}$$

which is an alternative, explicit equation to estimate x_2, where C is an empirical constant.

The empirical constant B was estimated for **Equation S5** by using the experimental values obtained in this work for TAL solubility in water at various temperatures and maximizing the coefficient of determination, R^2. This resulted in a value of 6303.3 J·mol^{-1} for B in **Equation S5**, with R^2 maximized to a value of 0.988 (**Figure S3A**).

In addition, we found from experimental measurement of TAL solubility in water (**Table S4**) that the value of x_2 is 5.03 *10^{-4} mol-TAL·mol-solution^{-1} at 0°C and 1.87 * 10^{-2} mol-TAL·mol-solution^{-1} at 93°C. As x_2 is monotonic against T, this means that within a temperature range of $[0℃, 93℃]$ the value of $(1 - x_2)^2$ will have a maximum of approximately 0.999 (at 0°C) and a minimum of approximately 0.963 (at 93°C). Therefore, it can be reasonably assumed that $(1 - x_2)^2 \approx 1 \; \forall \, T \in [0℃, 93℃]$, enabling the use of **Equation S6**, which is an alternative, explicit equation to estimate x_2. The empirical constant C was estimated for **Equation S6** by using the experimental values obtained in this work for TAL solubility in water at various temperatures and maximizing the coefficient of determination, R^2. This resulted in a value of 6134.2 J·mol^{-1} for C in **Equation S3**, with R^2 maximized to a value of 0.983 (**Figure S3B**). The **Equation S6** was fit to the experimental data to provide a faster, analytical method for estimating TAL solubility in water with a similar accuracy to the slower, numerical methods enabled by **Equations S3** and **S5**. However, the **Equations S4**, **S5**, and **S6** were not used to estimate solubility for any biorefinery simulations. Rather, **Equation S3**, being associated with the highest goodness of fit (R^2 value of 0.992), was exclusively used to estimate TAL solubility for all simulations in this work.

S1.4. Estimating the pH of Simulated Streams

The following approach was used to estimate the pH of the fermentation broth, which contained a mixture of two weak triprotic acids, phosphoric acid (1.31 x 10^{-3} M in the baseline case) and citric acid (8.67 x 10^{-2} M in the baseline case). Triacetic acid lactone was not included as its dissociation constant (approximately 10$^{-5.14}$) was roughly three orders of magnitude smaller than the first dissociation constant of phosphoric acid (10$^{-2.15}$) and roughly two orders of magnitude smaller than the first dissociation constant of citric acid (10$^{-3.13}$).

The three dissociation constants $K_{a,1}$, $K_{a,2}$, $K_{a,3}$ of a triprotic acid H_3A can be represented as:

$$K_{a,1} = \frac{[H^+][H_2A^-]}{[H_3A]} \quad \text{(Equation S7)}$$

$$K_{a,2} = \frac{[H^+][HA^{2-}]}{[H_2A^-]} \quad \text{(Equation S8)}$$

$$K_{a,3} = \frac{[H^+][A^-]}{[HA^2-]} \quad \text{(Equation S9)}$$

Further, from mass balance, the initial concentration $[\mathrm{H_3A}]_0$ of the triprotic acid (before dissociation) can be estimated by:

$$[\mathrm{H_3A}]_0 = [\mathrm{H_2A^-}] + [\mathrm{HA^{2-}}] + [\mathrm{A^-}] \qquad \text{(Equation S10)}$$

From **Equations S4, S5, S6,** and **S7**, the concentration of protons in the aqueous solution of a single polyprotic acid can be estimated using the equation:

$$[\mathrm{H^+}] = \frac{K_w}{[\mathrm{H^+}]} + [\mathrm{H_3A}]_0 \frac{[\mathrm{H^+}]^2 K_{a,1} + 2[\mathrm{H^+}]K_{a,1}K_{a,2} + 3K_{a,1}K_{a,2}K_{a,3}}{[\mathrm{H^+}]^3 + [\mathrm{H^+}]^2 K_{a,1} + [\mathrm{H^+}]K_{a,1}K_{a,2} + K_{a,1}K_{a,2}K_{a,3}} \qquad \text{(Equation S11)}$$

Therefore, the concentration of protons $[\mathrm{H^+}]$ in the aqueous solution of two polyprotic acids H_3A and H_3B can be estimated using the equation:

$$[\mathrm{H^+}] = \frac{\mathrm{K_w}}{[\mathrm{H^+}]} + [\mathrm{H_3A}]_0 \frac{[\mathrm{H^+}]^2 K_{a,1} + 2[\mathrm{H^+}]K_{a,1}K_{a,2} + 3K_{a,1}K_{a,2}K_{a,3}}{[\mathrm{H^+}]^3 + [\mathrm{H^+}]^2 K_{a,1} + [\mathrm{H^+}]K_{a,1}K_{a,2} + K_{a,1}K_{a,2}K_{a,3}} + [\mathrm{H_3B}]_0 \frac{[\mathrm{H^+}]^2 K_{b,1} + 2[\mathrm{H^+}]K_{b,1}K_{b,2} + 3K_{b,1}K_{b,2}K_{b,3}}{[\mathrm{H^+}]^3 + [\mathrm{H^+}]^2 K_{b,1} + [\mathrm{H^+}]K_{b,1}K_{b,2} + K_{b,1}K_{b,2}K_{b,3}}$$
(Equation S12)

Finally, the pH can be estimated using the equation:

$$pH = -\log_{10}[\mathrm{H^+}] \qquad \text{(Equation S13)}$$

We used $[\mathrm{H_3A}]_0$ = 1.31 x 10^{-3} M for the baseline concentration of phosphoric acid and $[\mathrm{H_3B}]_0$ = 8.67 x 10^{-2} M for the baseline concentration of citric acid in the fermentation broth. Values for dissociation constants for phosphoric acid ($K_{a,1}$ = $10^{-2.16}$, $K_{a,2}$ = $10^{-7.21}$, $K_{a,3}$ = $10^{-12.32}$) and citric acid ($K_{b,1}$ = $10^{-3.13}$, $\mathrm{K_{b,2}}$ = $10^{-4.76}$, $K_{b,3}$ = $10^{-6.40}$) were obtained from the literature.[19] Using equations (SK) and (SL), we estimated the pH of the fermentation broth to be 2.10 with the baseline citric acid concentration, and 2.94 with no citric acid in the broth (**Figure S7**). For the purposes of estimating pH, we assumed ideal activity coefficients for both acids. To model acid neutralization on addition of sodium hydroxide, we assumed complete dissociation of sodium hydroxide (a strong base) and that all phosphoric acid was neutralized first (as it was the stronger of the two), following which citric acid was neutralized.

After enough sodium hydroxide was added to the stream to completely neutralize both phosphoric and citric acids (i.e., above a pH of 7.0; **Figures S6** and **S12**), we continued to assume all further added sodium hydroxide was dissociated completely (i.e., $[\mathrm{OH^-}]$ increases by the same amount as $[\mathrm{NaOH}]_0$), and pH of the stream for pH>7.0 was estimated using the equation:

$$pH = 14 + \log_{10}[\mathrm{OH^-}] \qquad \text{(Equation S14)}$$

S1.5. Details of Biorefinery Techno-Economic Analysis and Life Cycle Assessment

The biorefinery was modeled as an nth plant design (i.e., it is assumed a successful industry has been established with mature technologies). All costs and prices shown are presented in 2019 U.S. dollars. The baseline federal corporate tax rate set to 21% (the current U.S. rate as of 2017[20]), with a range of 15–28% assumed in the uncertainty analysis to account for possible changes.[21,22] The minimum product selling price (MPSP, $\$\cdot kg^{-1}$) of TAL needed to achieve a net present value of zero was estimated based on a targeted annual internal rate of return (10% for the baseline, with a uniform range of 8–12 % assumed based on values proposed in previous sugarcane biorefinery TEAs[23–25]). A full list of baseline values and distributions for parameters included in the uncertainty analysis (e.g., TEA parameters including raw material prices, biorefinery annual operating time, TAL production capacity, federal corporate tax rate, and targeted internal rate of return) is included in **Tables S6** and **S7**.

Final characterization and discussion of environmental impacts focused on two impact categories—cradle-to-grave carbon intensity (CI; quantified as 100-year global warming potential, GWP_{100}) and fossil energy consumption (FEC)—which were selected to enable comparisons with

results from the literature and based on their relevance to policies and legislation.[26,27] The impact assessment methodology used for CI was the Intergovernmental Panel on Climate Change (IPCC) 2013[28] (from the Fifth Assessment Report, which the US 45Z Clean Fuel Production Credit and the Paris Agreement both still rely on[29]) and that used for FEC was the Cumulative Energy Demand method in ecoinvent 3.8.[30] The sum of carbon fixed in the feedstock and in the input corn steep liquor is equal to the sum of carbon in the product (TAL) and in the biogenic portion of direct waste emissions from the biorefinery. To focus on the biorefinery, we assumed the end-of-life impacts associated with the product (TAL) as well as non-gaseous wastes (e.g., unconsumed and non-combusted sugars and insoluble lignin in the brine, accounting for <0.05% of the CI) would be exclusively from passive oxidation of all carbon into CO_2. Impacts resulting from infrastructure construction were excluded to be consistent with the U.S. renewable fuel standard (RFS).[31] Life cycle inventory data were collected from ecoinvent 3.8 and some unit impacts were gathered from GREET 2020,[30,32] and their sources were noted in the script.[10]

S1.6. Selection of Uncertainty Distributions

We assigned uniform distributions for parameters for which literature values were lacking and triangular distributions for parameters with strong literature support for most probable values and ranges. In order to reflect the uncertainties in the parameters, a tiered system was used to determine the types and ranges of uncertainty distributions (specific distributions and references listed in **Table S4**):

1. For feedstock GWP_{100} and FEC characterization factors, we applied uniform distributions due to limited data availability.
2. For parameters with more than 20 data points, we applied triangular distributions and used 5th, 50th, and 95th percentiles as the lower bound, most probable (also baseline), and upper bound.
3. For parameters with 3-20 data points (e.g., natural gas and acetate prices), we applied triangular distributions and used the minimum, average, and maximum literature value as the lower bound, most probable (also baseline), and upper bound.
4. For parameters where the distributions were from literature, but the literature did not note the origin of the value (e.g., boiler efficiency), we used the same baseline value as the literature and chose a uniform distribution with the minimum and maximum values from the literature distribution as the lower and upper bounds.
5. For parameters with 1-2 data points (e.g., diammonium phosphate unit price), we applied a uniform distribution with the smaller and greater values from the literature as the lower and upper bounds where 2 data points were available. For parameters with only one data point available, we applied a large uniform distribution from 80% (minimum) to 120% (maximum) of the available value. In case 120% of the baseline value exceeded theoretical limits to the parameter's value (e.g., for yield, recovery), we instead capped the maximum value of the uniform distribution to the corresponding theoretical limit, and set a minimum value of the uniform distribution to be lower than the baseline by the same amount as the difference between the baseline and the maximum (i.e., "mirroring" the maximum value).
6. For fermentation TAL yield, titer, and productivity, as well as fermentation cell mass yield and citric acid yield, we applied a large uniform distribution from 80% (minimum) to 120% (maximum) of the reported values to characterize the implications of uncertainty in the current fermentation performance of the microbial candidate *Y. lipolytica*. Similarly, for TAL solubility in water, we applied a large uniform distribution from 80% (minimum) to 120%

(maximum) of the values from the one-parameter van Laar activity-based solubility model fit to the solubility-temperature data points we obtained experimentally in this work to characterize the implications of uncertainty in TAL solubility in the fermentation broth, which contains other impurities.

7. For crystallization time, we chose a large uniform distribution of 2–14 hours (8 hours at the baseline) to characterize the uncertainty in TAL crystallization kinetics at industrially relevant scales. Similarly, for the fermentation aeration rate safety factor, we chose a large uniform distribution of 0.5–2.0 (1.0 at the baseline) to characterize the uncertainty in volumetric oxygen transfer coefficients (kLa) in industrial fermentation vessels.
8. As a wide range of values for TAL ring-opening decarboxylation conversion was observed experimentally (potentially due to non-uniform heating by the thermocouple), we chose a uniform distribution of 4.63–34.0 mol% conversion based on the minimum and maximum values observed experimentally in this study.

For all uncertainty analyses, samples were generated for each of the parameters listed in **Tables S6** and **S7** using Latin hypercube sampling to reduce the number of simulations needed to yield reproducible results. This approach to defining probability density functions was based on those used in prior works on sugarcane and lignocellulosic biorefineries.[33–35]

S1.7. Facilities

Wastewater streams are treated by anaerobic digestion, producing biogas for on-site heat and electricity. The wastewater treatment facility includes an internal circulation reactor, aerobic membrane bioreactor, polishing filter, belt thickener, biogas upgrading, reverse osmosis, and sludge centrifuge for dewatering; the treated wastewater is reused in other processes. Solid residuals from the feed sugarcane juicing and TAL separation processes (containing filter cake and bagasse from the sugarcane juicing process, and cellular debris from the fermentation process), and biogas from anaerobic digestion are sent to the boiler and burned to reduce waste and recover energy. Given that energy recovered from the combustion can be used to generate enough steam to meet the total heating demand of the biorefinery, the excess steam is diverted to a turbogenerator, which produces enough electricity to satisfy the on-site electricity demand. Excess electricity is assumed to be sold back to the grid. Cooling demand is managed by the cooling tower and chilled water and brine system facilities, which use electricity to regenerate cooling utilities (cooling water, chilled water, and chilled brine). Water usage throughout the biorefinery is managed by the process water center unit, which supplements water needs (in addition to water recycled from the reverse osmosis unit) with fresh makeup water. The product (solid TAL, at least 89 wt% purity, 25 °C) is stored in a stainless steel 316 tank prior to sale.

To reduce total heating and cooling utility demands, a heat exchanger network (HXN) is implemented. The physical location of the HXN facility is distributed across the relevant units (rather than having a centralized configuration) to minimize heat lost during pumping. For each simulation, an initial pinch analysis is conducted to identify the pinch temperature and minimum energy targets, and viable pairs of hot and cold streams throughout the system are then matched on either side of the identified pinch. Subsequently, these matches are rigorously modeled using vapor-liquid equilibrium and simulated as process heat exchangers to calculate the offset to the utility demands of the original heat exchangers. Design details of all heat exchangers in the HXN are available online.[10] A list of all major units comprising the biorefinery, including facilities, is included in **Table S2**.

Section S2. Supplementary Results

S2.1. Breaking Down Process Contributions to System Costs and Environmental Impacts

Across the biorefinery, the components with the largest equipment purchase and installation costs were the boiler and turbogenerator (47% [43−51%] of the biorefinery's equipment purchase and installation costs) and the fermentation process (30% [27−34%]; **Figure 4A**). The boiler and turbogenerator were especially capital-intensive due to the high amount of solid waste (specifically sugarcane bagasse, centrifuged cellular materials after fermentation, and filter cake after juice clarification) and biogas (from anaerobic digestion of liquid waste streams) diverted to the boiler for combustion, and due to the resulting production of excess electricity by the turbogenerator. The high contribution of the boiler and turbogenerator to the total installed equipment cost is consistent with the results from other published TEAs of sugarcane biorefineries (e.g., 48−55%[24,36]). This suggests integrating the production of TAL with other bioproducts may improve financial viability by decreasing the contribution of the boiler and turbogenerator to the total installed equipment cost due to economies of scale. The high capital cost of the fermentation process was mainly due to the low baseline productivity (0.12 $g \cdot L^{-1} \cdot h^{-1}$), which resulted in long residence times for the stainless-steel fermentation and seed reactors). The capital cost associated with the wastewater treatment process was also high because of the dilute, high-volume waste streams generated by the biorefinery. Despite the long residence times (2−14 h; 8 h for the baseline) assumed for TAL crystallization (step 3 in **Figure 2B**), the installed equipment cost associated with the separation process did not excessively influence the total installed equipment cost (7% [5−10%]; **Figure 4A**). The baseline biorefinery's total capital cost was 287 MM$ and included: (i) direct costs (171 MM$) from the installed equipment cost (158 MM$), warehouse (3 MM$), site development (7 MM$), and additional piping (3 MM$); (ii) indirect costs (103 MM$); and (iii) working capital (14 MM$; a detailed breakdown of the total capital cost is included in **Table S8**).

The annual operating cost (27.2 $MM\$ \cdot y^{-1}$ [20.7−36.9 $MM\$ \cdot y^{-1}$]) was the sum of the annual material cost (37.3 $MM\$ \cdot y^{-1}$ [27.4−49.5 $MM\$ \cdot y^{-1}$]), the fixed operating cost (7.3 $MM\$ \cdot y^{-1}$ [6.4−8.6 $MM\$ \cdot y^{-1}$]; breakdown provided in **Table S9**), the cost of natural gas for product drying (0.183 $MM\$ \cdot y^{-1}$ [0.123−0.273 $MM\$ \cdot y^{-1}$]), and the revenue from the sale of excess co-produced electricity (17.6 $MM\$ \cdot y^{-1}$ [10.1−25.7 $MM\$ \cdot y^{-1}$]; **Figure 4A**). For the annual material cost, the largest contributors were the purchase of feed sugarcane (58% [50−66%]) and the purchase of materials required for the fermentation process (42% [27−59%]; **Figure 4A**), the latter's cost stemming primarily from the purchase of sodium acetate (13% [10−17%]), sodium hydroxide (12% [9−14%]), diammonium phosphate (9% [4−16%]), and corn steep liquor (8% [4−11%]). Other processes accounted for much smaller shares (tabulated data available online[10]). The separation process recovered 72% [60−87%] of TAL present in the fermentation broth despite the loss of TAL due to ring-opening decarboxylation and in the crystallization supernatant (in steps 2 and 5, respectively, in **Figure 2B**). The large contributions to the annual material cost from purchasing feed sugarcane and sodium acetate indicated improvements in the fermentation yield over the current state-of-technology (approximately 40.5% of theoretical) may substantially improve biorefinery economics.

We modeled the separation process to allow splitting the liquid supernatant stream (from step 4 in **Figure 2B**) into a recycled stream and a stream diverted to wastewater treatment (WWT). TAL recovery initially increased slightly with greater recycling (e.g., baseline TAL recovery was 72% with zero recycling, increasing to 73% when 53% of the supernatant was recycled; **Figure S2**). However, additional supernatant recycling actually decreased TAL recovery (e.g., TAL recovery was 61% when 95% of the supernatant was recycled) because the additional supernatant recycling led to dilute streams with low TAL recovery in crystallization. We found MPSP, CI, and FEC were lowest at zero split (i.e., at the baseline, with no supernatant recycling; **Figures**

S2B,C,D) as recycling the dilute supernatant stream necessitated larger equipment and higher cooling and power utility inputs.

The biorefinery's total heating and cooling duties (82 GJ·h^{-1} [60−122 GJ·h^{-1}] and 150 GJ·h^{-1} [94−254 GJ·h^{-1}], respectively) during operation were the sums of the heating and cooling utility demands from individual process areas (a total heating demand of 87 GJ·h^{-1} [64−129 GJ·h^{-1}] and cooling demand of 155 GJ·h^{-1} [98−260 GJ·h^{-1}]) and offsets from the heat exchanger network (23 GJ·h^{-1} [4−7 GJ·h^{-1}] and 21 GJ·h^{-1} [3−6 GJ·h^{-1}], respectively). The leading users of heating utilities were found to be the fermentation (38% [35−47%], due mainly to evaporative concentration of the clarified sugarcane juice to achieve the designated fermentation yield-titer combination), feedstock juicing (37% [29−40%], to heat the juice prior to phosphoric acid treatment and dissolved air removal), separation (23% [19−26%], from heating to dissolve TAL in the broth; step 1 in **Figure 2B**), and wastewater treatment (1% [0−7%], to maintain a temperature of 35°C in the internal circulation reactor, as the influent waste stream primarily comprised the supernatant from crystallization performed at 1°C). The leading users of cooling utilities were found to be the fermentation process (49% [36−66%]), the boiler (29% [15−40%]), and the separation process (19% [12−29%], energy basis; **Figure 4A**). About 13%, 11%, and 2% of the biorefinery's cooling demand at the baseline was associated with maintaining a temperature of 28°C in the fermentation reactors (as reported for *Y. lipolytica*[37]; **Table S3**), cooling compressed air for aeration during fermentation, and cooling the clarified sugarcane juice prior to fermentation, respectively. The cooling demand of the separation process is entirely associated with TAL crystallization (step 3 in **Figure 2B**). Other processes accounted for much smaller shares of heating and cooling utility demands (tabulated data available online[10]).

Regarding electricity consumption during operation (11.6 MW [7.0−23.2 MW]), the primary contributors were the fermentation process (53% [38−69%], primarily from air compression for aeration during fermentation) and cooling utility regeneration facilities (15% [7−23%], primarily from chilled water regeneration; **Figure 4A**). Feedstock juicing (9% [5−14%]) was also an important contributor (**Figure 4A)**, as it included large reactors that required continuous stirring. The boiler (9% [4−14%]), separation process (7% [4−12%]), and storage and other facilities (2% [1−4%]) accounted for smaller shares (**Figure 4A**; tabulated data available online[10]). The turbogenerator produced enough electricity to satisfy the biorefinery's power demand consistently (i.e., in 100.0% of simulations), with excess electricity (58.3 MW [31.7−90.9 MW]) sold at a price of \$0.07·kWh^{-1} (baseline values and uncertainty distributions for electricity unit price and all parameters included in the uncertainty analysis are detailed in **Table S6**).

Regarding the biorefinery's carbon intensity (CI) and fossil energy consumption (FEC), feedstock (sugarcane) growth, harvesting, and transportation accounted for 41% [34−50%] and 19% [15−25%] of positive (i.e., detrimental) contributions to the CI and FEC, respectively (**Figures 4B,C**). Contributions to CI and FEC from the acquisition of materials required for fermentation were also significant; namely, corn steep liquor (32% [20−40%] and 37% [25−45%], respectively), sodium hydroxide (6% [5−8%] and 13% [10−18%], respectively), acetate (4% [3−6%] and 18% [16−23%], respectively), and diammonium phosphate (4% [2−7%] and 9% [5−13%], respectively). The large uncertainties in the contributions of corn steep liquor and diammonium phosphate acquisition stemmed primarily from the uncertainties attributed to the loading requirements of those materials in the fermentation media to satisfy microbial nitrogen and phosphorus requirements (uniformly distributed between 41.7−102 g·L^{-1} for corn steep liquor and 5.55−14.9 g·L^{-1} for diammonium phosphate; further explained in **Section S1.1**). Direct non-biogenic GHG emissions accounted for 12% [10−15%] of positive (i.e., detrimental) contributions to CI. Acquisition of natural gas for product drying (0.18% [0.11−0.29%] of CI and 3.8% [2.2−5.6%] of FEC) and other materials (0.06% [0.05−0.07%] of CI and 0.09% [0.07−0.12%] of FEC, from

acquisition of phosphoric acid for sugarcane juice clarification and of caustic materials for wastewater treatment) accounted for much smaller shares (tabulated data available online[10]).

S2.2. Global Sensitivity Analysis Results

Of the 30 parameters included in the uncertainty analysis, the parameters to which MPSP was most sensitive (i.e., |Spearman's ρ| ≥ 0.10 and *p*-value<0.05) were TAL ring-opening decarboxylation conversion during separation (Spearman's ρ of 0.53), targeted annual internal rate of return (0.35), biorefinery annual operating days (-0.34), fermentation TAL yield (-0.33), fermentation TAL titer (-0.26), feed sugarcane unit price (0.19), desired annual TAL production capacity (-0.19), *Y. lipolytica* cell mass yield (-0.17), fermentation aeration rate (0.15), centrifuge recovery of crystallized TAL (-0.14), fermentation TAL productivity (-0.13), diammonium phosphate unit price (0.12), boiler efficiency (-0.12), and turbogenerator efficiency (-0.12; full sensitivity analysis results are presented in **Figure S8**).

The parameters to which CI was most sensitive were fermentation corn steep liquor loading (Spearman's ρ of 0.50), fermentation TAL titer (-0.48), boiler efficiency (-0.39), fermentation *Y. lipolytica* cell mass yield (-0.30), turbogenerator efficiency (-0.30), fermentation aeration rate (0.21), and TAL ring-opening decarboxylation conversion during separation (0.15). Biorefinery FEC was most sensitive to the boiler efficiency (Spearman's ρ of -0.40), fermentation TAL titer (-0.40), fermentation TAL yield (0.40), fermentation corn steep liquor loading (0.31), TAL ring-opening decarboxylation conversion during separation (-0.30), turbogenerator efficiency (-0.30), fermentation *Y. lipolytica* cell mass yield (-0.29), fermentation aeration rate (0.22; full sensitivity analysis results are presented in **Figure S8**).

S2.3. System Sustainability Implications of Targeted Fermentation Improvements

The following targeted improvements were explored to illustrate the potential benefits of additional microbial conversion research and development: (i) fermentation TAL yield increase from 40.5% (0.19 $g \cdot g^{-1}$) to 73.0% of theoretical (0.34 $g \cdot g^{-1}$, comparable to the reported yield of 0.39 $g \cdot g^{-1}$ using *E. coli* to produce adipic acid,[38] another 6-carbon metabolite with low solubility in water); and (ii) fermentation TAL titer increase from 35.9 $g \cdot L^{-1}$ to 68.0 $g \cdot L^{-1}$ (equal to the reported adipic acid titer of 68.0 $g \cdot L^{-1}$ achieved using *E. coli*[39]). If these two targets are achieved while maintaining the same annual TAL production capacity as the baseline (13385 metric ton $TAL \cdot y^{-1}$; baseline values and uncertainty distributions detailed in **Tables S6** and **S7**), the biorefinery's MPSP would decrease to \$3.14$\cdot kg^{-1}$ [\$2.55−3.96$\cdot kg^{-1}$]. The resulting MPSP of TAL would be lower than the maximum viable price range as a sorbic acid feedstock (\$5.99−7.74$\cdot kg^{-1}$) in 100.0% of simulations (**Figure S9A**). The increase in yield would result in an increase to baseline FEC (from -17.2 [-55.6−22.9] $MJ \cdot kg^{-1}$ to (-4.1 [-26.3−14.4] $MJ \cdot kg^{-1}$), with net displacement of fossil energy consumption (i.e., FEC<0) in a slightly decreased 61.8% of simulations (compared to 72.7% of simulations for the current state-of-technology; **Figure S9C**). However, the biorefinery's CI would decrease to 3.35 [1.93−4.82] kg CO_2-eq$\cdot kg^{-1}$, lower than the benchmark dimedone CI[40] (8.0 kg CO_2-eq$\cdot kg^{-1}$) in an increased 100.0% of simulations (compared to 89.9% of simulations for the current state-of-technology; **Figure S9B**). The significance of the fermentation TAL yield and titer for the biorefinery's financial viability and environmental benefits highlights the need for continued improvements in the performance of microbes engineered for biological TAL production.

Results from similar analyses conducted across fermentation performance at productivities lower (20% of baseline; **Figure S4**) and higher (500% of baseline; **Figure S5**) than baseline are included. At the baseline yield-titer combination, decreasing productivity to 20% of the baseline increased CI by 4.9% (to 5.57 kg CO_2-eq$\cdot kg^{-1}$), FEC by 19.5% (to -13.9 $MJ \cdot kg^{-1}$), and MPSP by 60.5% (to \$7.39$\cdot kg^{-1}$). Increasing productivity to 500% of the baseline did not significantly affect

CI or FEC (decrease of 0.9% and 3.9%, respectively), and decreased MPSP by 13.0% (to \$4.00·kg^{-1}; **Figure S5**). The installed equipment cost of the fermentation process was 47.0 MM\$ at the baseline productivity, 179.3 MM\$ at 20% of the baseline productivity, and 18.5 MM\$ at 500% of the baseline productivity, corresponding to 29.6%, 61.6%, and 14.3% of the total installed equipment costs of those biorefineries, respectively.

S2.4. Market-Driven Capacity Expansion and Operating Schedule Considerations

The sensitivity analysis performed for the baseline scenario highlighted the significance of operating time and TAL production capacity on the economics of the biorefinery. While the baseline TAL production capacity was 13385 metric ton TAL·y^{-1} (comparable to the growth projected in the U.S. sorbic acid demand between 2020−2030, comparable to the amount by which the 2019 U.S. demand exceeded the 2019 U.S. production capacity, and equivalent to 50% of the 2019 U.S. sorbic acid demand[41]), there is significant potential for larger production capacities to meet current and projected U.S. and global demands for a range of potential products for which TAL can serve as a feedstock (including sorbic acid,[42,43] polydiketoenamine plastics,[40] acetylacetone,[42] pogostone,[44] katsumadain,[45] and penicipyrone,[46] among others). Further, there is large uncertainty in the operating schedule for sugarcane biorefineries (e.g., 120−200 annual operating days[23–25]), and there exists the potential for biorefineries to additionally accept sweet sorghum as a feedstock (as the composition is similar to sugarcane[23]) to significantly increase biorefinery operating time (e.g., to 240 annual operating days[23]). To quantify the economic implications of alternative biorefinery operating times and TAL production capacities, we simulated and evaluated the biorefinery across the production-operation space (i.e., 6400 combinations of biorefinery operating time and TAL production capacities; **Figure S6**).

Across the evaluated combinations, MPSP benefited from increased operating times and TAL production capacities. Although the sensitivity analysis performed for the baseline indicated biorefinery operating time may be more critical than TAL production capacity for the MPSP, this was primarily due to the relatively large uncertainty attributed to the operating time (120−240 annual operating days; baseline values, distributions, literature references, and Spearman's ρ for all parameters are detailed in **Table S6** and **Figure S8**). The relative impact on the MPSP from improvements to operating time against that from improvements to TAL production capacity depended on the location in the production-operation space. In general, improvements to TAL production capacity were more impactful at lower operating time values and improvements to operating time were more impactful at high production capacities. These trends stem from tradeoffs between the biorefinery's capital and operating costs. Across the production-operation space, while higher production capacities increased both capital and operating costs (**Figure S6 B,C**), the increase in capital cost diminished with increasing production capacities due to economies of scale while revenue from product sales increased linearly, resulting in benefits to the MPSP by increasing production capacity (**Figure S6A**). Higher operating times resulted in decreased capital (as the desired annual TAL production capacity could be achieved with smaller equipment; **Figure S6B**) and annual operating costs (**Figure S6C**) across the production-operation space. The annual operating cost was the sum of fixed and variable operating costs, and the relative significance of the former (which increased with improvements in production capacity) diminished with increasing variable operating costs (which increased with both operating time and production capacity improvements). The benefits to total capital investment, annual operating cost, and MPSP by increasing the operating time were therefore greatest at points with high production capacity and low operating time (**Figure S6**).

To further improve financial viability over the baseline scenario, an alternative strategy requiring the same total capital investment as the baseline (287 MM\$) would be to accept feedstocks having composition similar to sugarcane (e.g., sweet sorghum[23]) that can be harvested during at least

two months (May and September) when sugarcane in the southern U.S. is not harvested.[23] This additional feedstock could enable an increase in annual operating time to 240 days and an increase in TAL production capacity by about 33.5% to 17869 metric ton TAL·y^{-1} (requiring approximately an additional 208000 metric tons of feed sweet sorghum annually, well below annual sweet sorghum capacities of 229000−533000 metric tons for sugarcane-sorghum biorefineries proposed in a previous study[23]). This strategy would reduce the MPSP by \$0.78·kg^{-1} to \$3.82·kg^{-1}, which is \$2.17·kg^{-1} below the lowest end of the maximum viable price range as a feedstock for sorbic acid production (**Figure S6A**).

Further, maintaining an annual operating time of 240 days and investing additional capital to increase TAL production capacity would significantly improve the biorefinery's financial viability, even without any improvements over the baseline fermentation or separation performance. For example, if TAL production capacity were increased to the minimum amounts needed to meet the 2019 U.S. sorbic acid demand (23800 metric tons) or the projected 2030 U.S. demand (34550 metric tons) by producing at least 26770 and 38864 metric tons of TAL, respectively, the MPSP would be further reduced to \$3.52·kg^{-1} and \$3.31·kg^{-1} TAL, respectively (**Figure S6A**). This highlights the potential to further improve the financial viability of TAL production through capacity expansion and improved operating schedules by integrating multiple feedstocks.

S2.5. Exploring Potential Separation Improvements by pH Control

In the separation process designed for the baseline biorefinery, the broth from fermentation is initially heated to dissolve all TAL (step 1 in **Figure 2B**) to later enable crystallization of TAL largely free of cellular debris and other solid impurities (e.g., a baseline TAL product stream of 91 wt% purity, with 5.0 wt% water and 3.2 wt% cellular debris as the main impurities). However, we experimentally observed that heating aqueous TAL solutions resulted in ring-opening decarboxylation of TAL to acetylacetone (e.g., 20.9 mol% conversion at the baseline, with a uniform range of 4.63–34.0 mol% assumed for the uncertainty analysis; **Table S6**). Ring-opening decarboxylation of TAL with high conversion and selectivity has been reported to occur without the presence of a catalyst when aqueous solutions of TAL are subject to high temperature and pressure (e.g., 373 K under 21 bar He maintained for 4 hours), producing acetylacetone and CO_2.[42,47] The ring-opening decarboxylation of TAL is reportedly initiated by the reversible keto–enol tautomerization of TAL, followed by nucleophilic addition of water to the lactone carbonyl, both steps that require the presence of protons (H^+) in solution.[47] Therefore, we computationally explored the sustainability implications of potential strategies to mitigate the ring-opening decarboxylation of TAL by controlling the pH of the stream during heating (i.e., step 1 in **Figure 2B**) by adding a base, sodium hydroxide. The pH of the stream was 6.5 in the baseline case after the base added for pH control neutralized acids present in the broth, namely phosphoric acid (1.31×10^{-3} M in the baseline case) added during feedstock pretreatment and citric acid (8.67×10^{-2} M in the baseline case) produced during fermentation (method for estimating pH detailed in **Section S1.4**). Although we observed a TAL ring-opening decarboxylation conversion of 4.63–34.0 mol% of the theoretical by heating aqueous solutions of TAL for 1 hour to various temperatures (**Table S5**), this does not necessarily mean the reaction attained equilibrium within that time, and a higher total residence time may be needed for heating, centrifugation, and cooling (steps 1–3 in **Figure 2B**). We designed and simulated the biorefinery across 3600 potential combinations of TAL ring-opening decarboxylation conversion and pH maintained (**Figure S7**).

The biorefinery's FEC was lower at higher values for TAL ring-opening decarboxylation conversion (e.g., baseline FEC of -17.2 MJ·kg^{-1} reduced to -51.5 MJ·kg^{-1} at 50.0 mol% TAL ring-opening decarboxylation conversion; **Figure S7C**). This was because a lower conversion of TAL to acetylacetone resulted in lower-energy waste streams being diverted to the anaerobic digestion for biogas production, which resulted in lower excess electricity production. The offset from

excess electricity production decreases the total positive FEC by 119% at the baseline (resulting in a net negative value for FEC; **Figure 4C**). However, the biorefinery's FEC excluding offsets from co-produced electricity consistently benefited from decreases in TAL loss by ring-opening decarboxylation (**Figure S12C**).

Both MPSP and CI benefited from decreases in TAL ring-opening decarboxylation. The potential minima of MPSP and CI were $3.65·kg^{-1} and 4.54 kg CO_2-eq·kg^{-1}, respectively (**Figure S7A,B**). Although increasing the pH maintained necessitated greater amounts of sodium hydroxide, increasing pH maintained to 11.5 would not substantially influence MPSP, CI, or FEC (increased by $0.01·kg^{-1}, 0.01 kg CO_2-eq·kg^{-1}, and 0.15 MJ·kg^{-1}, respectively. Increasing pH maintained from 11.5 to 12.5, however, necessitated substantially greater amounts of sodium hydroxide and resulted in corresponding increases to MPSP (by $0.33·kg^{-1}), CI (by 0.11 kg CO_2-eq·kg^{-1}), and FEC (by 1.45 MJ·kg^{-1}), respectively. Although the added sodium hydroxide may react with carbon dioxide produced by ring-opening decarboxylation to form sodium carbonate, potentially further reducing the CI, this was not considered. The relative insensitivity of MPSP and CI to pH requirements below 11.5 raises the possibility of developing strategies to mitigate TAL loss by ring-opening decarboxylation. For example, if a pH of 11.0 maintained by sodium hydroxide addition were sufficient to decrease TAL ring-opening decarboxylation conversion during separation from 20.9 mol% (baseline) to 4.8 mol%, the MPSP would be reduced to $3.84·kg^{-1} ($0.77·kg^{-1} lower than the baseline), the CI would be reduced to 4.69 kg CO_2-eq·kg^{-1} (0.62 kg CO_2-eq·kg^{-1} lower than the baseline), and FEC would increase to -7.4 MJ·kg^{-1} (9.82 MJ·kg^{-1} higher than the baseline).

Section S3. Supplementary Figures

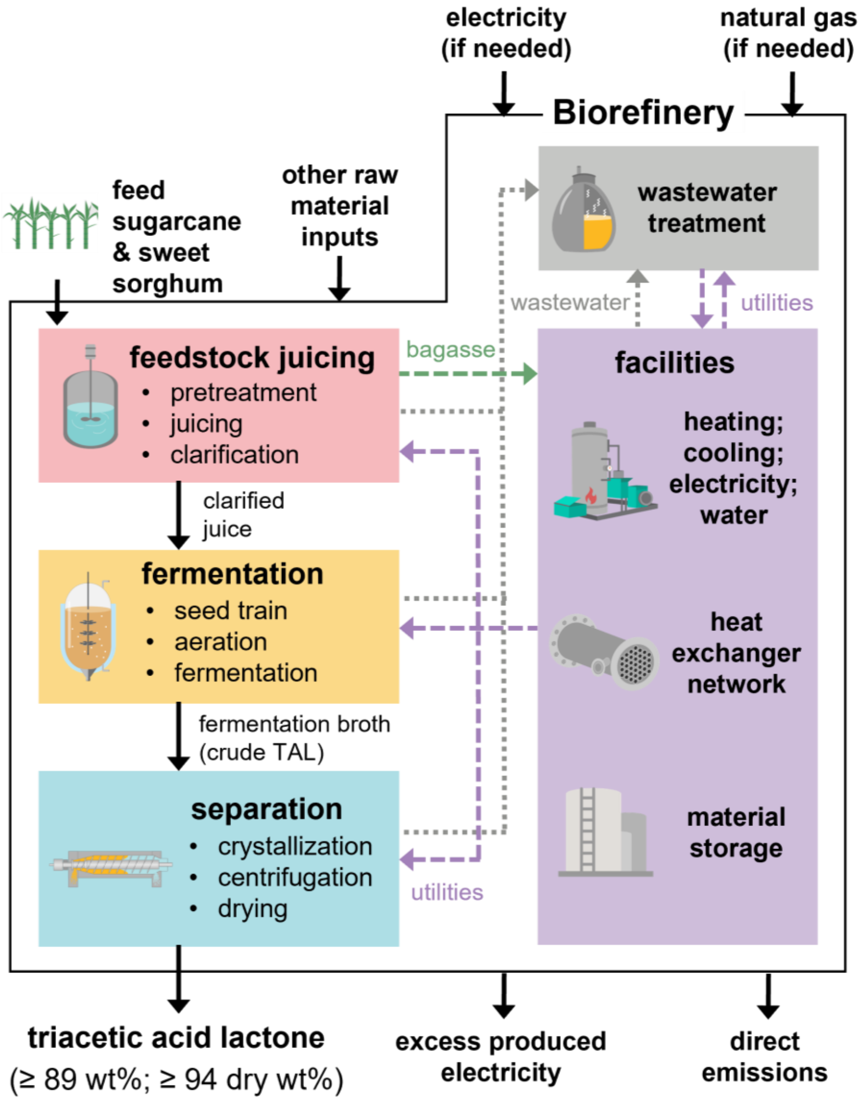

Figure S1. Simplified overview of the designed biorefinery. The solid black line labeled 'Biorefinery' represents the system boundary. For carbon intensity (CI) as 100-year global warming potential (GWP_{100}), end-of-life emissions were included (2.09 kg CO_2-eq·kg^{-1}, assumed to be entirely from passive oxidation of TAL to CO_2).

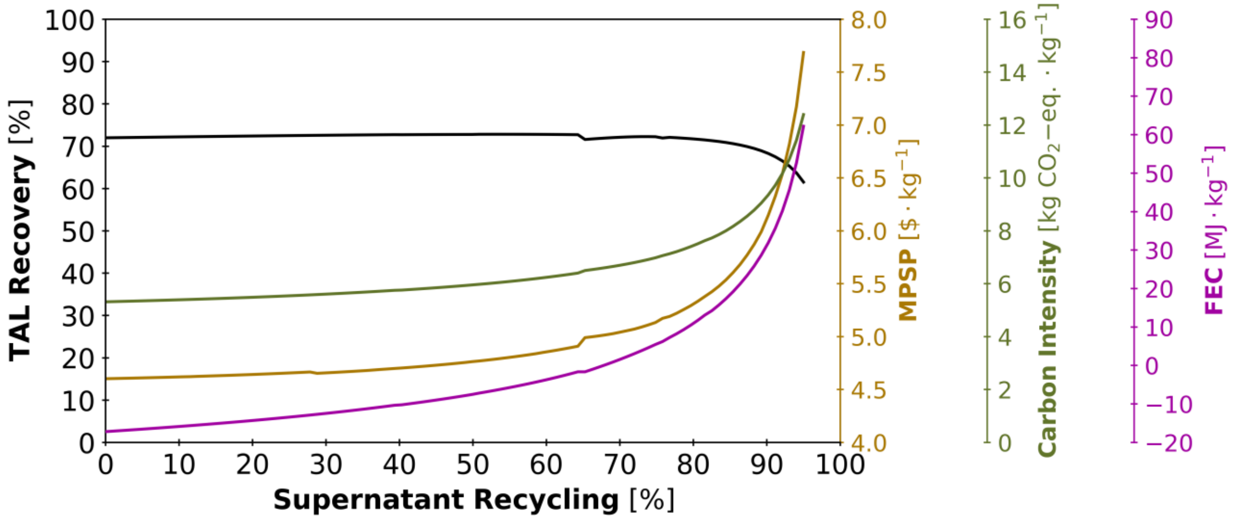

Figure S2. TAL recovery, MPSP, GWP_{100}, and FEC (*y*-axes) at 100 alternative values for supernatant recycling (*x*-axis) from 0–95% (inclusive) at baseline TAL ring-opening decarboxylation conversion (20.9 mol%).

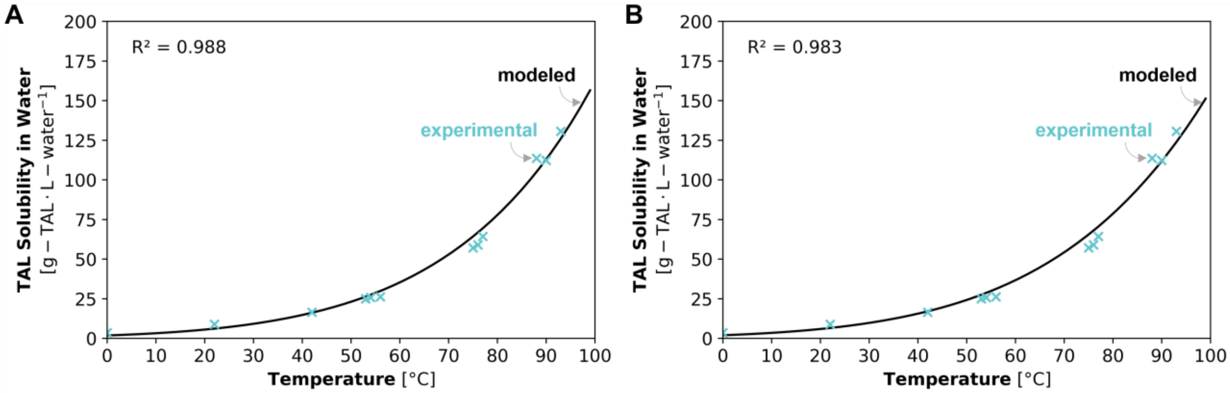

Figure S3. Implicit [A] and simplified explicit [B] forms of the one-parameter Margules activity-based model for TAL solubility in water described by Equations S5 and S6, respectively.

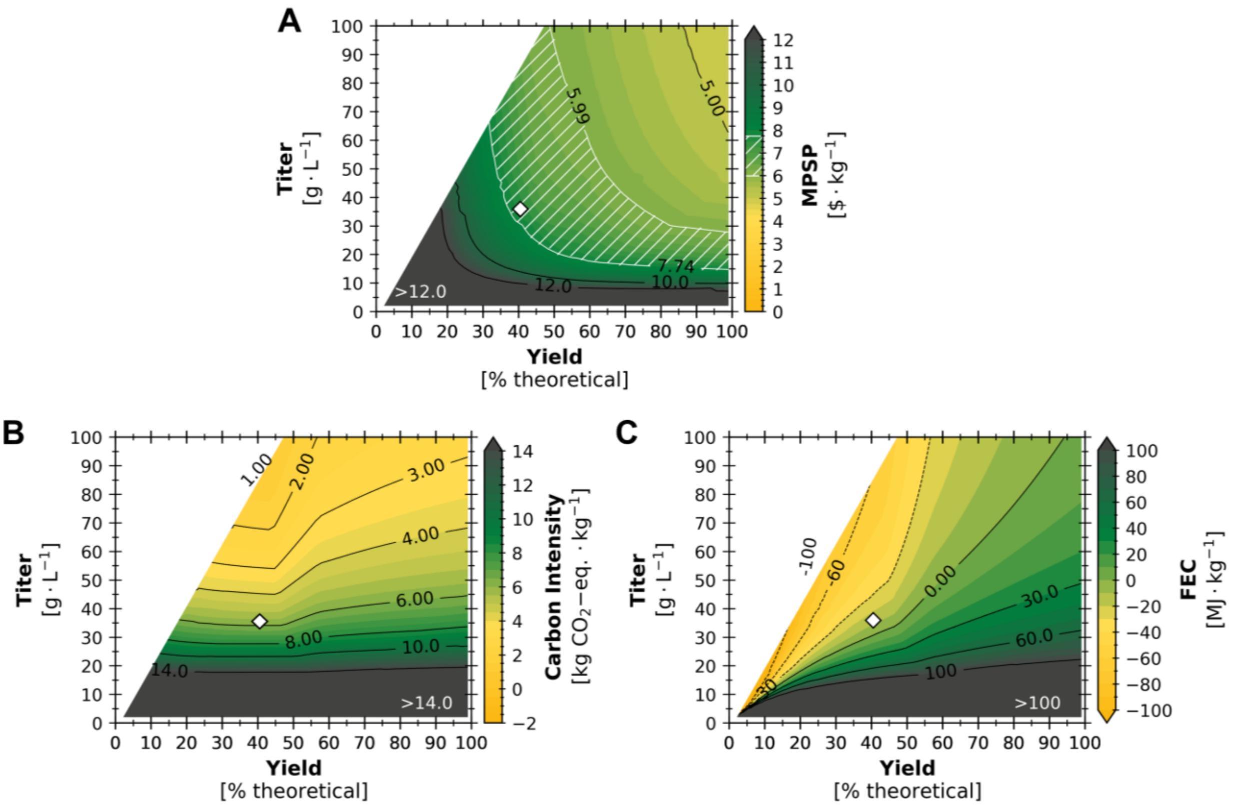

Figure S4. (A) Minimum product selling price (MPSP), (B) 100-year global warming potential (GWP_{100}), and (C) fossil energy consumption (FEC) of the produced TAL across fermentation TAL yield (*x*-axis) and titer (*y*-axis) at one-fifth the baseline productivity ($0.024\ g\cdot L^{-1}\cdot h^{-1}$). For a given point on the figure, the *x-axis* value representa overall fermentation TAL yield (as the percent of maximum theoretical yield of TAL on glucose, sucrose, and acetate, where the maximum theoretical yield is assumed to be $0.467\ g\cdot g\text{-glucose-eq.}^{-1}$ and $0.467\ g\cdot g\text{-acetic-acid-eq.}^{-1}$), the *y-axis* value represents the titer, and the color represents MPSP, GWP_{100}, or FEC. The white region to the upper left of each plot represents infeasible yield-titer combinations (described in **Section S1.1**). The maximum viable TAL price range is represented (in A) by hatching with white diagonal lines between $\$5.99-7.74\cdot kg^{-1}$. The baseline yield-titer combination (represented by diamonds) constitutes a yield of 40.5% theoretical and a titer of $35.9\ g\cdot L^{-1}$.

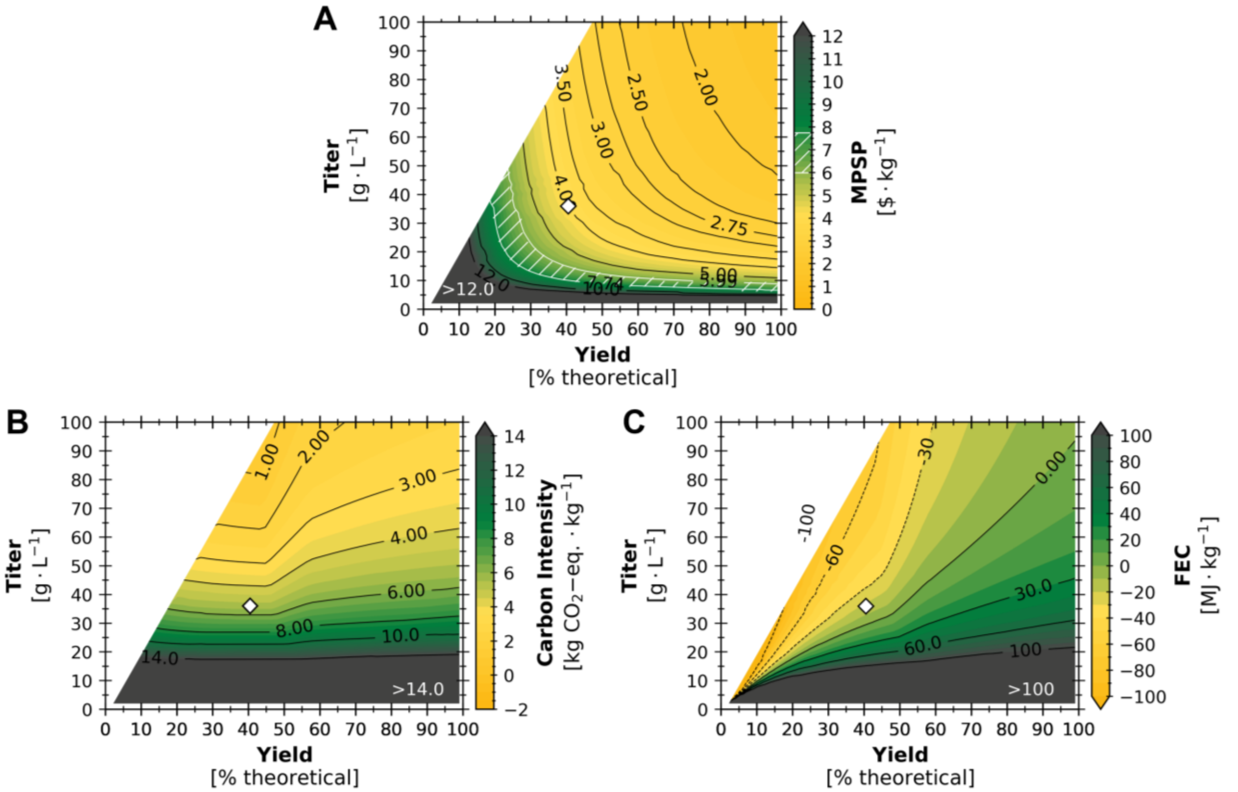

Figure S5. (A) Minimum product selling price (MPSP), (B) 100-year global warming potential (GWP_{100}), and (C) fossil energy consumption (FEC) of the produced TAL across fermentation TAL yield (*x*-axis) and titer (*y*-axis) at five times the baseline productivity (0.600 $g{\cdot}L^{-1}{\cdot}h^{-1}$). For a given point on the figure, the *x-axis* value representa overall fermentation TAL yield (as the percent of maximum theoretical yield of TAL on glucose, sucrose, and acetate, where the maximum theoretical yield is assumed to be 0.467 g·g-glucose-eq.$^{-1}$ and 0.467 g·g-acetic-acid-eq.$^{-1}$), the *y-axis* value represents the titer, and the color represents MPSP, GWP_{100}, or FEC. The white region to the upper left of each plot represents infeasible yield-titer combinations (described in **Section S1.1**). The maximum viable TAL price range is represented (in A) by hatching with white diagonal lines between \$5.99−7.74·kg^{-1}. The baseline yield-titer combination (represented by diamonds) constitutes a yield of 40.5% theoretical and a titer of 35.9 $g{\cdot}L^{-1}$.

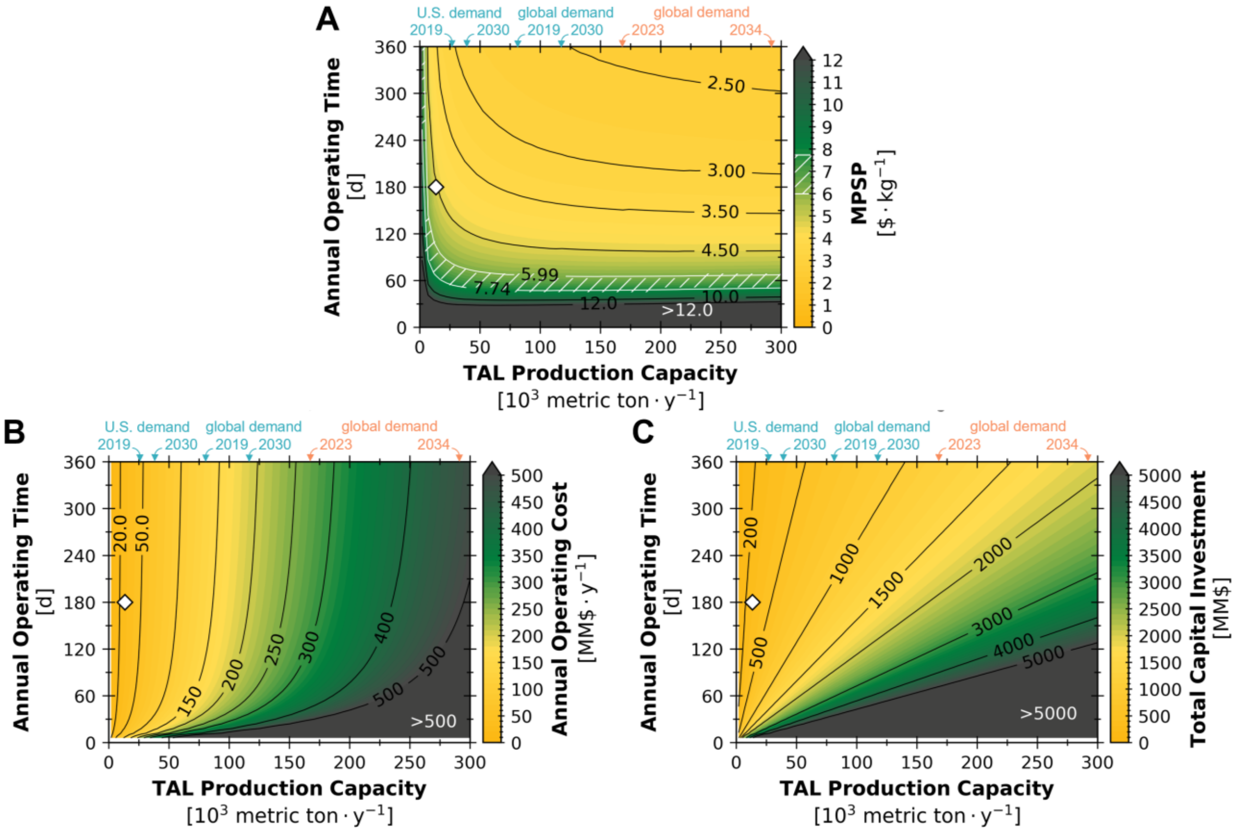

Figure S6. (A) Minimum product selling price (MPSP) of TAL, (B) total capital investment, and (C) annual operating cost across TAL production capacity (*x*-axes) and biorefinery annual operating time (*y*-axes). Diamond markers represent the baseline scenario, corresponding to a TAL production capacity of 13385 metric ton TAL·y^{-1} (comparable to the growth projected in the U.S. sorbic acid demand between 2020−2030, comparable to the amount by which the 2019 U.S. demand exceeded the 2019 U.S. production capacity, and equivalent to 50% of the 2019 U.S. sorbic acid demand[41]) and an annual operating time of 180 days (within the 120–200 operating days previously estimated for sugarcane biorefineries in the southern U.S. based on typical harvest periods and maximum storage times,[23–25] and well below the potential operating time of 240 days enabled by additionally accepting sweet sorghum[23]). Markers in the top *x*-axes indicate TAL production capacities equivalent to U.S. and global demands for sorbic acid reported (*2019*, *2023*) or projected (*2030*, *2034*) for specified years by Transparency Market Research[41] (blue) or ChemAnalyst[48] (orange). The maximum viable TAL price range as a feedstock for sorbic acid production is represented (in A) by hatching with white diagonal lines between \$5.99−7.74·kg^{-1}. The benchmark of \$10·kg^{-1} as a feedstock replacing dimedone for polydiketoenamine plastics production is represented (in A) as a standard contour line.

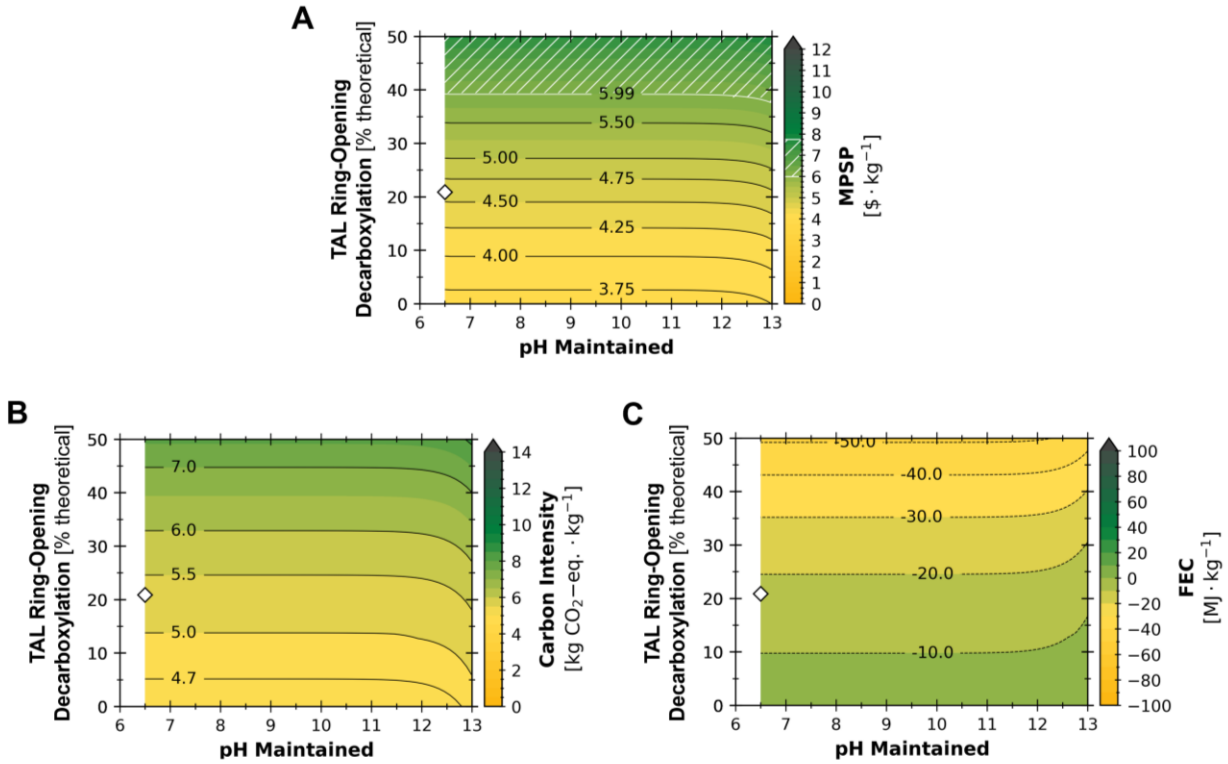

Figure S7. (A) Minimum product selling price (MPSP), (B) life cycle carbon intensity (CI), and (C) fossil energy consumption (FEC) of the produced TAL across potential ring-opening decarboxylation conversion of TAL to acetylacetone during heating (step 1 in the separation process; *x*-axes) and potential pH maintained by sodium hydroxide addition (*y*-axes). The maximum viable TAL price range as a sorbic acid feedstock is represented (in A,B) by hatching with white diagonal lines between $5.99−7.74·kg^{-1}. The benchmark ($10·kg^{-1}) for TAL replacing dimedone as a feedstock for polydiketoenamines is not represented here as all MPSP values (in A) are lower than this benchmark. Diamond markers represent the baseline (.9% TAL ring-opening decarboxylation conversion, pH of 6.5). The infeasible region (below the baseline pH of 6.5) represents pH values lower than can be achieved without acid addition.

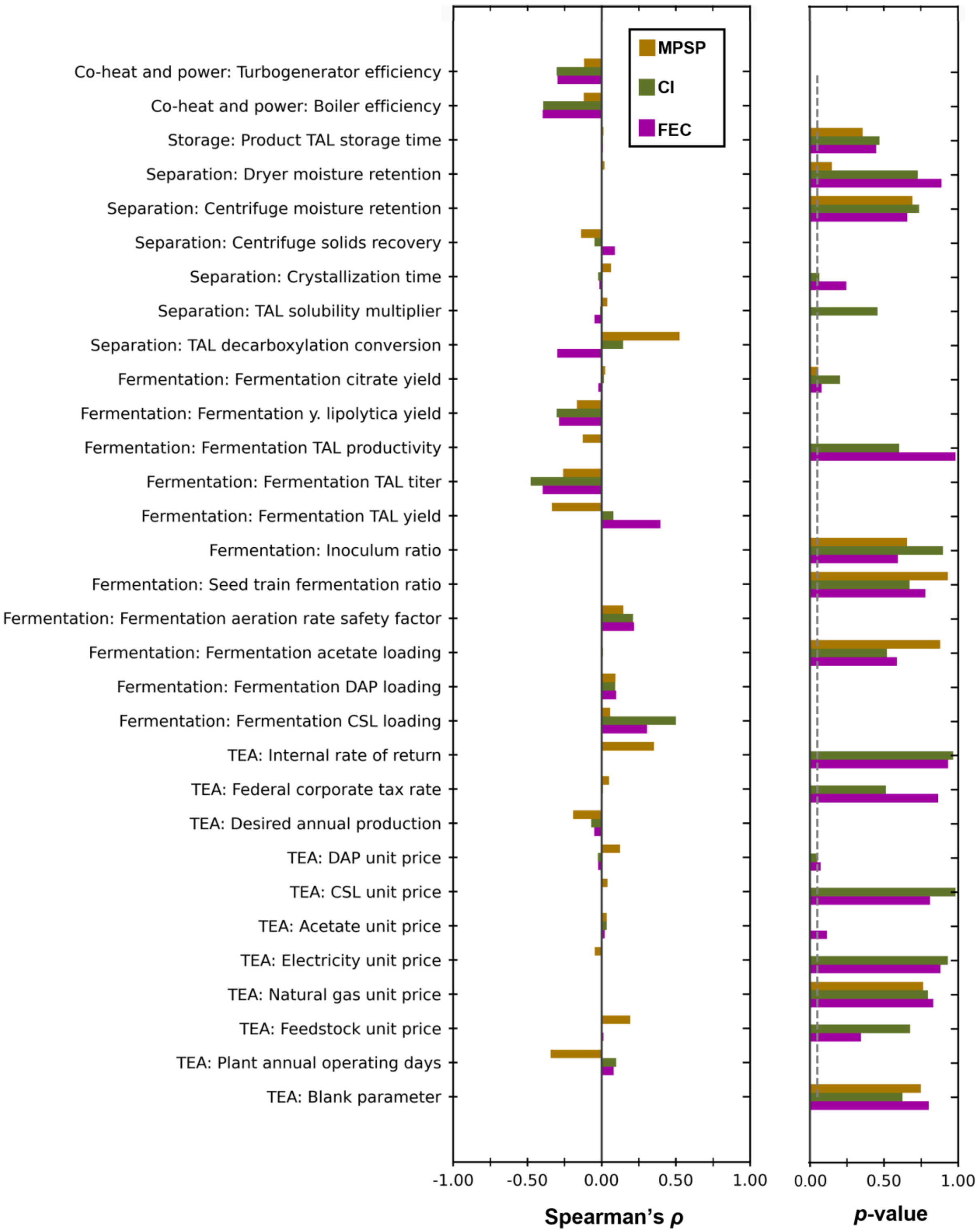

Figure S8. Sensitivity analysis results for the biorefinery in the *current* scenario as Spearman's rank order correlation coefficients (Spearman's ρ; *x*-axis, left; from -1.00 to 1.00) and corresponding *p*-values (*x*-axis, right; from 0.00 to 1.00) for minimum product selling price (MPSP), carbon intensity (CI), and fossil energy consumption (FEC), with respect to each of 30 parameters included in the uncertainty analysis (*y*-axes). The vertical dashed gray line represents a *p*-value of 0.05.

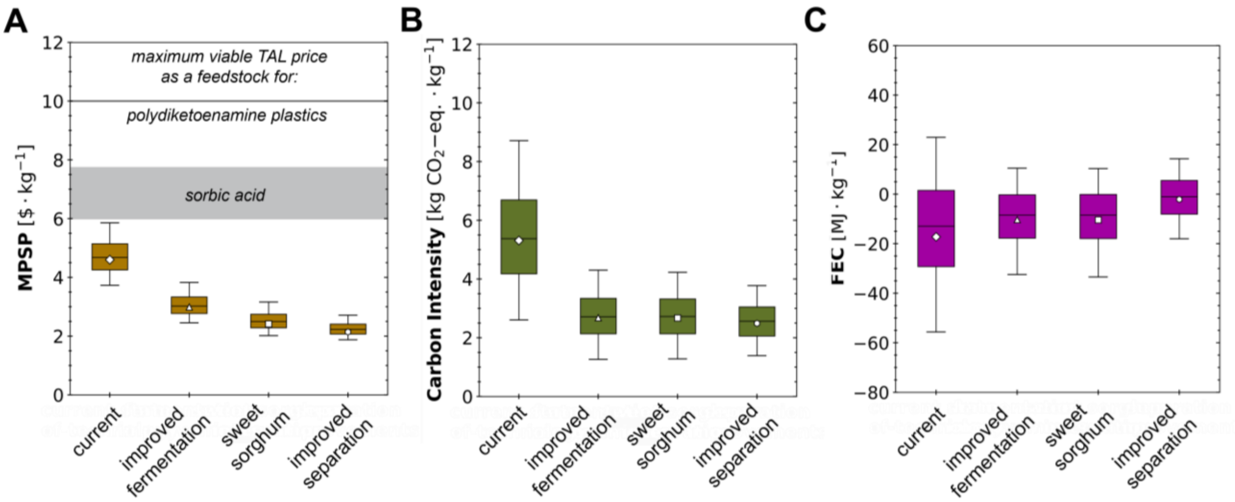

Figure S9. System sustainability for the scenario with improvements to fermentation TAL yield (to 68.0% of theoretical) and TAL titer (to 76.0 g·L[1]), represented by diamonds and stacked bar charts. Uncertainties (box-and-whisker plots) and breakdowns (stacked bar charts) for (A) minimum product selling price (MPSP), (B) carbon intensity (CI) as 100-year global warming potential, and (C) fossil energy consumption (FEC) per kg of TAL produced via fermentation of glucose and acetate by *Y. lipolytica*. Results are shown for four scenarios: the current state-of-technology (*current*, same as in Figure 4, shown here for comparison); improvements to fermentation TAL yield (to 73.0% theoretical) and titer (to 68.0 $g \cdot L^{-1}$; *improved fermentation*) relative to *current*; increase in annual operating time (to 240 days) and TAL production capacity (to 17689 metric $ton \cdot y^{-1}$) relative to *improved fermentation* by integrating sweet sorghum (*sweet sorghum*); and decreasing TAL loss by ring-opening decarboxylation (to 4.8 mol%) relative to *sweet sorghum* by adding sodium hydroxide (to maintain a pH of 11.0; *improved separation*). Whiskers, boxes, and the middle line represent $5^{th}/95^{th}$, $25^{th}/75^{th}$, and 50th percentiles, respectively, from 6000 Monte Carlo simulations in each scenario. Diamond (*current*), triangle (*improved fermentation*), square (*sweet sorghum*), and hexagon markers (*improved separation*) represent baseline values for each scenario. The shaded gray regions show the maximum viable price range for TAL as a dimedone replacement to produce polydiketoenamine plastics ($\$10 \cdot kg^{-1}$; the market price for dimedone)[40] and as a feedstock for sorbic acid ($\$5.99-7.74 \cdot kg^{-1}$; based on the market price range for sorbic acid of $\$6.74-8.71 \cdot kg^{-1}$).[41,49] Values and distributions with literature references for all parameters are detailed in Tables S6 and S7. Tabulated data breaking down capital and material costs, heating and cooling duties, electricity consumption, GWP_{100}, and FEC are available online.[10]

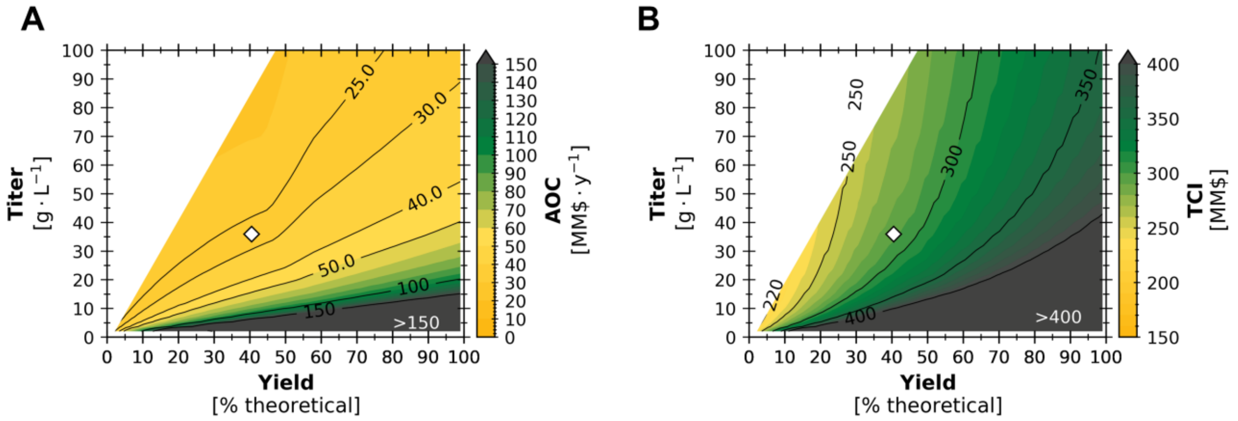

Figure S10. (A) Annual operating cost (AOC) and (B) total capital investment (TCI) for the biorefinery across fermentation TAL yield (*x*-axis) and titer (*y*-axis) at the baseline productivity (i.e., 0.12 g·L^{-1}·h^{-1}). For a given point on the figure, the *x-axis* value representnal overall fermentation TAL yield (as the percent of maximum theoretical yield of TAL on glucose, sucrose, and acetate, where the maximum theoretical yield is assumed to be 0.467 g·g-glucose-eq.$^{-1}$ and 0.467 g·g-acetic-acid-eq.$^{-1}$), the *y-axis* value represents the titer, and the color represents MPSP, GWP_{100}, or FEC. The white region to the upper left of each plot represents infeasible yield-titer combinations (described in **Section S1.1**). The baseline yield-titer combination (represented by diamonds) constitutes a yield of 40.5% theoretical and a titer of 35.9 g·L^{-1}.

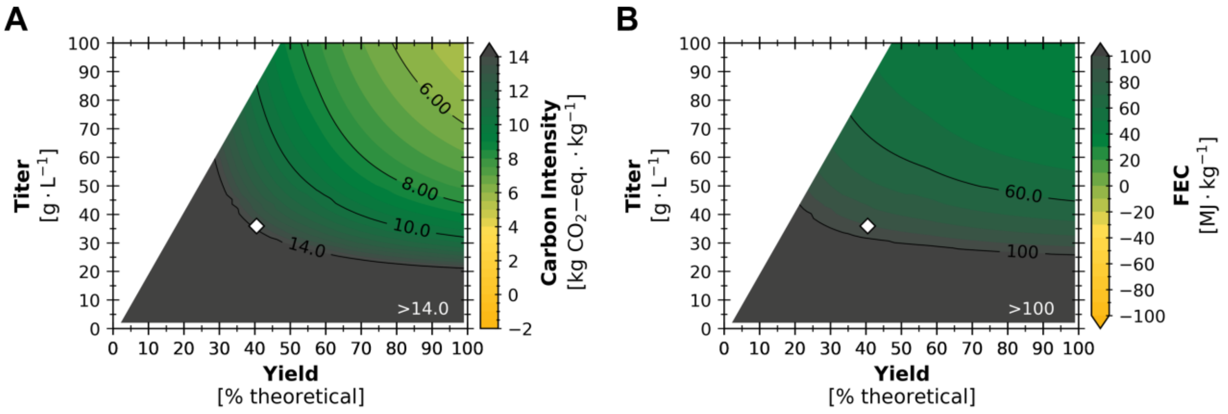

Figure S11. (A) 100-year global warming potential (GWP_{100}), and (B) fossil energy consumption (FEC) of the produced TAL across fermentation TAL yield (*x*-axis) and titer (*y*-axis) at the baseline productivity (i.e., 0.120 g·L^{-1}·h^{-1}) excluding offsets to these environmental impacts from the production of excess electricity. For a given point on the figure, the *x-axis* value represents overall fermentation TAL yield (as the percent of maximum theoretical yield of TAL on glucose, sucrose, and acetate, where the maximum theoretical yield is assumed to be 0.467 g·g-glucose-eq.$^{-1}$ and 0.467 g·g-acetic-acid-eq.$^{-1}$), the *y-axis* value represents the titer, and the color represents MPSP, GWP_{100}, or FEC. The white region to the upper left of each plot represents infeasible yield-titer combinations (described in **Section S1.1**). The baseline yield-titer combination (represented by diamonds) constitutes a yield of 40.5% theoretical and a titer of 35.9 g·L^{-1}.

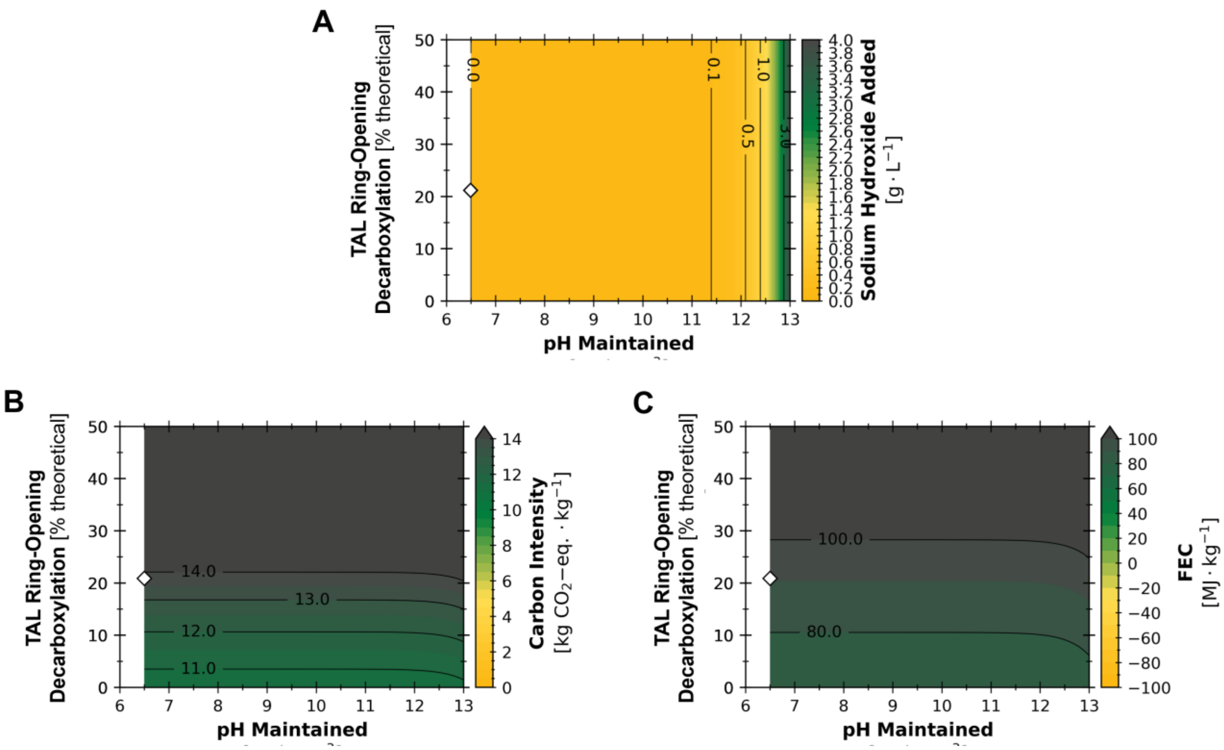

Figure S12. (A) Sodium hydroxide added and (B) carbon intensity (CI) and (C) FEC impacts excluding offsets from co-produced electricity across ring-opening decarboxylation conversion of TAL to acetylacetone during heating (step 1 in the separation process; *x*-axis) and pH maintained by sodium hydroxide addition (*y*-axis). The diamond marker represents the baseline (20.9% TAL ring-opening decarboxylation conversion, pH of 6.5). The infeasible region (below the baseline pH of 6.5) represents pH values lower than can be achieved without acid addition.

Section S4. Supplementary Tables

Table S2. Assumed composition of feed sugarcane modeled to be consistent with Cortes-Pena et al.[50]

**Component**	**Composition (wt%)**
Water	70
Glucose	1.21
Sucrose	13.7
Lignin	3.28
Ash	0.6
Cellulose	6.12
Hemicellulose	3.61
Solids	1.5

Table S2. List of major units and equipment included in the biorefinery.

Process	ID	Unit	Equipment	Sources
feedstock juicing	U201	Crushing mill	crushing mill	[50]
	U202	Conveying belt	conveying belt	[50]
	M201	Mixer	mixer	BioSTEAM
	S201	Vibrating screen	vibrating screen	[50]
	T202	Storage tank	tank	BioSTEAM
	H201	Heat exchanger	floating head	BioSTEAM
	T203	Mix tank	tank	BioSTEAM
	P201	Pump	pump, motor	BioSTEAM
	T204	Mix tank	tank	BioSTEAM
	T205	Mix tank	tank	BioSTEAM
	P202	Pump	pump, motor	BioSTEAM
	M202	Mixer	mixer	BioSTEAM
	H202	Heat exchanger	floating head	BioSTEAM
	T206	Mix tank	tank	BioSTEAM
	C201	Clarifier	clarifier	BioSTEAM
	C202	Rotary vacuum filter	vessels, vacuum system - liquid-ring pump, oil seal, vacuum system	[50]
	P203	Pump	pump, motor	BioSTEAM
fermentation	F301	Multi-effect evaporator	condenser, mixer, vacuum system, evaporators	BioSTEAM
	F301 P	Pump	pump, motor	BioSTEAM
	H301	Heat exchanger	floating head	BioSTEAM
	M304	Mix tank	tank	BioSTEAM
	M304 H	Heat exchanger	double pipe	BioSTEAM
	R302	Batch co fermentation	heat exchangers, reactors, agitators, cleaning in place, recirculation pumps	design based on [5] and Table S3, costs based on [5]
	R303	Seed train	pumps, agitators, reactors, heat exchangers	design based on [5] and Table S3, costs based on [5]
	T301	Seed hold tank	pump, agitator, tank	BioSTEAM
	K301	Isothermal compressor	compressors	BioSTEAM
	V301	Valve	valve	BioSTEAM

Table S2. List of major units and equipment included in the biorefinery (continued).

Process	ID	Unit	Equipment	Sources
separation	M401	Liquids mixing tank	turbine agitator, vertical pressure vessel, platform and ladders	BioSTEAM
	H401	Heat exchanger	floating head	BioSTEAM
	M402	Liquids mixing tank	turbine agitator, vertical pressure vessel, platform and ladders	BioSTEAM
	S401	Solids centrifuge	centrifuges	BioSTEAM
	F401	Multi-effect evaporator	condenser, mixer, vacuum system, evaporators	BioSTEAM
	F401 P0	Pump	pump, motor	BioSTEAM
	F401 P1	Pump	pump, motor	BioSTEAM
	C401	Crystallizer	crystallizer	BioSTEAM
	S402	Solids centrifuge	centrifuges	BioSTEAM
	F402	Drum dryer	drum dryer	BioSTEAM
	F403	Multi-effect evaporator	condenser, mixer, vacuum system, evaporators	BioSTEAM
	F403 P0	Pump	pump, motor	BioSTEAM
	F403 P1	Pump	pump, motor	BioSTEAM
	H420	Heat exchanger	double pipe	BioSTEAM
wastewater	M501	Mixer	mixer	BioSTEAM
	M502	Mixer	mixer	BioSTEAM
	U501	Internal circulation reactor	tank, heat exchangers effluent pump, sludge pump	BioSTEAM, [51]
	U502	Anaerobic membrane bioreactor	tank, membrane, pumps, air pipes, blowers, degassing membrane, heat exchangers	BioSTEAM, [51]
	U503	Polishing filter	tank, packing polymers, pumps, degassing membrane, heat exchangers	BioSTEAM, [51]
	U504	Belt thickener	thickeners, effluent pump, sludge pump	BioSTEAM, [51]
	C501	Sludge centrifuge	centrifuges, effluent pump, sludge pump	BioSTEAM, [51]
	M503	Mixer	mixer	BioSTEAM
	M504	Mixer	mixer	BioSTEAM
	U505	Biogas upgrading	biogas upgrading unit	BioSTEAM, [51]
	U506	Reverse osmosis	evaporator, reactor	BioSTEAM, [51]

Table S2. List of major units and equipment included in the biorefinery (continued).

Process	ID	Unit	Equipment	Sources
facilities	M901	Mixer	mixer	BioSTEAM
	T601	CSL storage tank	tank	BioSTEAM
	T601 P	Pump	pump, motor	BioSTEAM
	T607	Sodium acetate storage tank	tank	BioSTEAM
	T607 P	Conveying belt	conveying belt	BioSTEAM
	T608	Acetylacetone storage tank	tank	BioSTEAM
	T608 P	Pump	pump, motor	BioSTEAM
	T609	DAP storage tank	tank	BioSTEAM
	T609 P	Pump	pump, motor	BioSTEAM
	T620	TALStorageTank	tank	BioSTEAM
	T620 P	Pump	pump, motor	BioSTEAM
	CWP802	Chilled water package	chilled water package	BioSTEAM
	BT701	Boiler turbogenerator	baghouse bags, boiler, deaerator, amine addition package, hot process water softener system, turbogenerator	BioSTEAM
	CT801	Cooling tower	cooling tower, cooling water pump	BioSTEAM
	CWP803	Chilled brine package	chilled brine package	BioSTEAM
	HXN1001	Heat exchanger network	heat exchangers	BioSTEAM
	CIP901	CIP Package	cip package	BioSTEAM
	ADP902	Air distribution package	plant air compressor, instrument air dryer, plant air reciever	BioSTEAM
	FWT903	Fire water tank	pump, tank	BioSTEAM
	PWC904	Process water center	tank, process water pump, makeup water pump	BioSTEAM

Table S3. Reference literature for fermentation process design.

**Microbe**	**Mode**	**Substrate**	**Substrate preparation**	**Time** [h]	**Temperature** [°C]	**pH control** [pH; agents]	**Dissolved oxygen** [saturation level]	**Final TAL yield** [$g \cdot g^{-1}$ C source[b]]	**Final TAL titer** [$g \cdot L^{-1}$]	**Mean TAL productivity** [$g \cdot L^{-1} \cdot h^{-1}$]	**Reference**
Escherichia	fed-batch	glycerol	artificial	n.r.[a]	n.r.[a]	n.r.[a]	n.r.[a]	0.102	2.06	n.r.[a]	[52]
Escherichia	fed-batch	glucose	artificial	168	36	7; NH4OH, H2SO4	20%	0.003	0.47	0.0028	[53]
Rhodosporidium	fed-batch	glucose, acetate	artificial	120	30	6; KOH, acetic acid	20%	0.074	28	0.24	[54]
Rhodosporidium	fed-batch	glucose, fructose, sucrose, acetate	oilcane juice; acetate supplementation	120	30	6; KOH, acetic acid	20%	0.089	23	0.19	[54]
Saccharomyces	fed-batch	glucose	artificial	120	n.r.[a]	6; NaOH, HCl	20%	0.13	2.2	0.018	[55]
Saccharomyces	fed-batch	glucose	artificial	48	30	n.r.[a]	n.r.[a]	0.16	1.6	0.033	[56]
Saccharomyces	fed-batch	ethanol	artificial	n.r.[a]	n.r.[a]	no pH control	n.r.[a]	n.r.[a]	5.2	n.r.[a]	[57]
Saccharomyces	fed-batch	glucose	artificial	168	30	5 for first 48 hours, 6 thereafter; NH4OH, H2SO4	20% for first 48 hours, 5% thereafter	0.049	0.37	0.0022	[53]
Saccharomyces	fed-batch	glucose	artificial	168	30	5 for first 48 hours, 6 thereafter; NH4OH, H2SO4	20% for first 48 hours, 5% thereafter	0.042	1.8	0.011	[53]
Saccharomyces	fed-batch	xylose, acetate	artificial	84	30	5; NaOH, HCl	n.r.[a]	n.r.[a]	23.91	0.29	[58]
Saccharomyces	fed-batch	glucose, xylose, acetate	switchgrass hemicellulose hydrolysate	115	30	5; NaOH, HCl	n.r.[a]	0.06745	3.55	0.0309	[58]

[a] Not reported.
[b] Mass of TAL divided by the total mass of sugars (sucrose, glucose, fructose, and xylose), ethanol, and acetate (acetic acid mass equivalent).

Table S3. Reference literature for fermentation process design (continued).

**Microbe**	**Mode**	**Substrate**	**Substrate preparation**	**Time** [h]	**Temperature** [°C]	**pH control** [pH; agents]	**Dissolved oxygen** [saturation level]	**Final TAL yield** [$g \cdot g^{-1}$ C source[b]]	**Final TAL titer** [$g \cdot L^{-1}$]	**Mean TAL productivity** [$g \cdot L^{-1} \cdot h^{-1}$]	**Reference**
Yarrowia	fed-batch	glucose, xylose, acetate	corn stover hydrolysate	190	30	5.5; KOH	n.r.[a]	n.r.[a]	21.6	0.114	[8]
Yarrowia	fed-batch	glucose	artificial	n.r.[a]	28	n.r.[a]	n.r.[a]	0.063	2.6	n.r.[a]	[2]
Yarrowia	fed-batch	xylose	artificial	n.r.[a]	28	n.r.[a]	n.r.[a]	n.r.[a]	2.9	n.r.[a]	[2]
Yarrowia	fed-batch	acetate	artificial	180	30	6.0; HCl	n.r.[a]	0.149	4.76	0.0264	[59]
Yarrowia	fed-batch	glucose, acetate	artificial	350	28	6.5; NaOH	50%	0.189	35.9	0.12	[1]
Yarrowia	batch	glucose	artificial	96	28	n.r.[a]	n.r.[a]	n.r.[a]	0.416	0.0043	[3]
Yarrowia	batch	glucose	artificial	80-96 hours	28	5.5; NaOH, NH4OH	21%	0.065	2.6	0.027	[3]

[a] Not reported.
[b] Mass of TAL divided by the total mass of sugars (sucrose, glucose, fructose, and xylose), ethanol, and acetate (acetic acid mass equivalent).

Table S4. Experimentally measured TAL solubility in water at various temperatures.

Temperature [°C]	TAL saturation concentration [g·L^{-1} water]
0	3.52E+00
22	8.92E+00
42	1.65E+01
53	2.50E+01
54	2.58E+01
56	2.62E+01
75	5.72E+01
76	5.90E+01
77	6.42E+01
88	1.14E+02
90	1.12E+02
93	1.31E+02

Table S5. Experimentally measured TAL ring-opening decarboxylation in water at various temperatures.

Temperature [°C]	Ring-Opening Decarboxylation Conversion of TAL [%]				
	Sample			Mean	Standard Error
	1	2	3		
30	1.04E+01	1.80E+01	1.34E+01	1.39E+01	2.22E+00
50	1.68E+01	3.40E+01	4.63E+00	1.85E+01	8.51E+00
80	1.47E+01	2.67E+01	2.61E+01	2.25E+01	3.89E+00

Table S6. List of parameters included in uncertainty and sensitivity analyses for the current state-of-technology (*current*). Note all chemical prices were converted to the TEA year, 2019 (conversion method detailed in the script[10]). Samples with >100% total conversion of glucose, sucrose, and acetate were capped to 100% total conversion with the following priority order for sample product yields: TAL, citric acid, and cell mass (*Y. lipolytica*).

Parameter name	Units	Base-line	Distribution Shape	Lower	Most Common	Upper	References
TEA							
plant annual operating days	d	180	triangular	120	180	240	from [24,50] for sugarcane (lower, most common, baseline) and integrated sweet sorghum (upper)
feedstock unit price	$·wet-kg^{-1}	0.0345	triangular	0.0276	0.0345	0.0414	baseline from [50]; bounds are baseline ±20%
natural gas unit price	$·kg^{-1}	0.2765	triangular	0.2163	0.2765	0.3321	minimum, mean, and maximum price during 2010-2019[60]
electricity unit price	$·kWh^{-1}	0.07	triangular	0.067	0.07	0.074	minimum, mean, and maximum price during 2010-2019[60]
sodium acetate unit price	$·kg-acetic-ac-d-eq.$^{-1}$	1.3691	triangular	0.931	1.369	1.808	minimum, mean, and maximum price in 2008[61]
CSL unit price	$·kg^{-1}	0.0747	triangular	0.0598	0.0747	0.0896	baseline from[33]; bounds are baseline ±20%
DAP unit price	$·kg^{-1}	0.6876	uniform	0.2827		1.0924	bounds from [5,61]; baseline is mean of bounds
desired annual TAL production	pure metric ton·y^{-}	13385	triangular	10708	13385	16062	baseline described in *System Description* in the *Methods* section of the manuscript; bounds are baseline ±20%
federal corporate tax rate	%	21.0	uniform	15.0		28.0	baseline from [20]; bounds based on [21,22]
internal rate of return	%	10.0	uniform	8.0		12.0	baseline for consistency with [5,50]; bounds are baseline ±20%
Fermentation							
fermentation CSL loading	g·L^{-1}	76.903	uniform	41.707		101.900	described in Section S1.1
fermentation DAP loading	g·L^{-1}	10.228	uniform	5.547		14.909	described in Section S1.1
fermentation sodium acetate loading	g-acetic-acid-eq.·L^{-1}	10	uniform	8		12	baseline from [1]; bounds are baseline ±20%
fermentation aeration rate safety factor	%	100	uniform	50		200	assumed
seed train fermentation ratio	%	95.0	uniform	90.0		100.0	baseline based on [5]; maximum is the theoretical limit (100%) and minimum mirrors the maximum
inoculum ratio	%	7.0	uniform	6.3		7.7	baseline based on [5]; bounds are ±10%
fermentation TAL yield	% theoretical	40.48	uniform	32.38		48.58	baseline from [1]; bounds are baseline ±20%

Table S6. List of parameters included in uncertainty and sensitivity analyses for the current state-of-technology, *current* (continued).

Fermentation *(continued)*							
fermentation TAL titer	$g \cdot L^{-1}$	35.9	uniform	28.72		43.08	baseline from [1]; bounds are baseline ±20%
fermentation TAL productivity	$g \cdot L^{-1} \cdot h^{-1}$	0.120	uniform	0.096		0.144	baseline from [1]; bounds are baseline ±20%
fermentation *Y. lipolytica* cell mass yield	$g\text{-cells-eq} \cdot g\text{-glucose-eq}^{-1}$	0.146	uniform	0.117		0.175	baseline described in Section S1.1; bounds are baseline ±20%
fermentation citric acid yield	% theoretical	8.86	uniform	7.08		10.6	based on concentrations reported in [1]
Separation							
TAL ring-opening decarboxylation conversion	% theoretical	20.9	uniform	4.63		34.0	range observed experimentally in this work; Table S5
TAL solubility multiplier	%	1	uniform	0.8		1.2	baseline is from equation (III); bounds are baseline ±20%
crystallization time	h	8	uniform	2		14	assumed
centrifuge solids recovery	%	95.0	uniform	90.0		100.0	baseline from [62]; maximum is the theoretical limit (100%) and minimum mirrors the maximum
centrifuge moisture retention	%	50.0	uniform	40.0		60.0	baseline from [62]; bounds are baseline ±20%
dryer moisture retention	%	5.00	uniform	4.00		6.00	baseline from [62]; bounds are baseline ±20%
Facilities							
product TAL storage time	h	168	triangular	134.4	168	201.6	baseline based on [5]; bounds are baseline ±20%
boiler efficiency	%	80.0	uniform	72.0		88.0	baseline from [63,64]; bounds are baseline ±10% based on [33]
turbogenerator efficiency	%	85.0	uniform	76.5		93.5	baseline from [62]; bounds are baseline ±10%

Table S7. List of parameters changed relative to the current state-of-technology (*current* scenario; Table S6) in uncertainty analyses for three scenarios: improvements to fermentation TAL yield (to 73.0% theoretical) and titer (to 65 $g\cdot L^{-1}$; *improved fermentation* scenario); increase in annual operating time (to 240 days) and TAL production capacity (to 17689 metric $ton\cdot y^{-1}$) relative to the *improved fermentation* scenario by integrating sweet sorghum (*sweet sorghum* scenario); and decreasing TAL loss by ring-opening decarboxylation (to 4.8 mol%) relative to *sweet sorghum* by adding sodium hydroxide (to maintain a pH of 11.0; *improved separation* scenario). Samples with >100% total conversion of glucose, sucrose, and acetate were capped to 100% total conversion with the following priority order for sample product yields: TAL, citric acid, and cell mass (*Y. lipolytica*).

Parameter name	Units	Base-line	Distribution Shape	Lower	Most Common	Upper	References
improved fermentation, *sweet sorghum*, and *improved separation*							
fermentation TAL yield	% theoretical	73.0	uniform	58.4		87.6	baseline assumed (see *Setting Targets for Fermentation Performance*); bounds are baseline ±20%
fermentation TAL titer	$g\cdot L^{-1}$	68.0	uniform	54.4		81.6	
sweet sorghum and *improved separation*							
plant annual operating days	d	240	triangular	150	240	300	from [24,50] for integrated sugarcane + sweet sorghum (baseline); bounds based on *current* (Table S6)
desired annual TAL production	pure metric $ton\cdot y^{-}$	17869	triangular	14295	17869	21443	baseline assumed (see *Market-Driven Capacity Expansion and Operating Schedule Considerations*); bounds are baseline ±20%
improved separation							
TAL ring-opening decarboxylation conversion	% theoretical	4.8	uniform	1.1		7.8	baseline assumed (see *Exploring Potential Separation Improvements by pH Control*); bounds based on *current* (Table S6)
pH maintained before heating	n/a	11	uniform	10		12	baseline assumed (see *Exploring Potential Separation Improvements by pH Control*); bounds are baseline ±10%

Table S8. Breakdown of the current state-of-technology (*current*) baseline biorefinery's total capital cost. *ISBL* and *OSBL* denote inside and outside battery limits, respectively.

**Cost category**	**Notes**	**Cost** [MM$]
Direct costs		
ISBL installed equipment cost	-	75.60
OSBL installed equipment cost	-	81.96
Warehouse	4.0% of ISBL	3.02
Site development	9.0% of ISBL	6.80
Additional piping	4.5% of ISBL	3.40
Total direct cost (TDC)	-	170.79
Indirect costs		
Pro-ratable costs	10.0% of TDC	17.08
Field expenses	10.0% of TDC	17.08
Construction	20.0% of TDC	34.16
Contingency	10.0% of TDC	17.08
Other indirect costs (start-up, permits, etc.)	10.0% of TDC	17.08
Total indirect cost (TIDC)	-	102.48
Fixed capital investment (FCI)	*TDC + TIDC*	273.27
Working capital (WC)	5.0% of FCI	13.66
Total capital investment (TCI)	*FCI + WC*	286.93

Table S9. Breakdown of the current state-of-technology (*current*) baseline biorefinery's fixed operating cost. *ISBL* and *OSBL* denote inside and outside battery limits, respectively.

**Cost category**	**Notes**	**Cost** [$MM\$\cdot y^{-1}$]
Labor salary	-	1.66
Labor burden	90% of labor salary	1.50
Maintenance	3.0% of FCI	2.27
Property insurance	0.7% of ISBL	1.91
Total fixed operating cost (FOC)	-	5.96

References

(1) Markham, K. A.; Palmer, C. M.; Chwatko, M.; Wagner, J. M.; Murray, C.; Vazquez, S.; Swaminathan, A.; Chakravarty, I.; Lynd, N. A.; Alper, H. S. Rewiring Yarrowia Lipolytica toward Triacetic Acid Lactone for Materials Generation. *Proceedings of the National Academy of Sciences of the United States of America* **2018**, *115* (9), 2096–2101. https://doi.org/10.1073/pnas.1721203115.

(2) Li, H.; Alper, H. S. Producing Biochemicals in Yarrowia Lipolytica from Xylose through a Strain Mating Approach. *Biotechnology Journal* **2020**, *15* (2). https://doi.org/10.1002/biot.201900304.

(3) Yu, J.; Landberg, J.; Shavarebi, F.; Bilanchone, V.; Okerlund, A.; Wanninayake, U.; Zhao, L.; Kraus, G.; Sandmeyer, S. Bioengineering Triacetic Acid Lactone Production in Yarrowia Lipolytica for Pogostone Synthesis. *Biotechnology and Bioengineering* **2018**, *115* (9), 2383–2388. https://doi.org/10.1002/bit.26733.

(4) CHEMANALYST. *North America Acetic Acid Market Analysis: Industry Market Size, Plant Capacity, Production, Operating Efficiency, Demand & Supply, End-User Industries, Sales Channel, Regional Demand, Company Share, Manufacturing Process, Foreign Trade, 2015-2032*; 2023. https://www.chemanalyst.com/industry-report/north-america-acetic-acid-market-2953 (accessed 2025-04-29).

(5) Humbird, D.; Davis, R.; Tao, L.; Kinchin, C.; Hsu, D.; Aden, A.; Schoen, P.; Lukas, J.; Olthof, B.; Worley, M.; Sexton, D.; Dudgeon, D. *Process Design and Economics for Biochemical Conversion of Lignocellulosic Biomass to Ethanol: Dilute-Acid Pretreatment and Enzymatic Hydrolysis of Corn Stover*; Technical Report NREL/TP-5100-47764; DOE: NREL, 2011. http://www.nrel.gov/docs/fy11osti/47764.pdf (accessed 2015-09-13).

(6) Shasaltaneh, M. D.; Moosavi-Nejad, Z.; Gharavi, S.; Fooladi, J. Cane Molasses as a Source of Precursors in the Bioproduction of Tryptophan by Bacillus Subtilis. *Iranian Journal of Microbiology* **2013**, *5* (3), 285.

(7) Niehus, X.; Casas-Godoy, L.; Rodríguez-Valadez, F. J.; Sandoval, G. Evaluation of *Yarrowia Lipolytica* Oil for Biodiesel Production: Land Use Oil Yield, Carbon, and Energy Balance. *Journal of Lipids* **2018**, *2018*, 1–6. https://doi.org/10.1155/2018/6393749.

(8) Cordova, L. T.; Lad, B. C.; Ali, S. A.; Schmidt, A. J.; Billing, J. M.; Pomraning, K.; Hofstad, B.; Swita, M. S.; Collett, J. R.; Alper, H. S. Valorizing a Hydrothermal Liquefaction Aqueous Phase through Co-Production of Chemicals and Lipids Using the Oleaginous Yeast Yarrowia Lipolytica. *Bioresource Technology* **2020**, *313*. https://doi.org/10.1016/j.biortech.2020.123639.

(9) Karbowiak, T.; Gougeon, R. D.; Alinc, J.-B.; Brachais, L.; Debeaufort, F.; Voilley, A.; Chassagne, D. Wine Oxidation and the Role of Cork. *Critical Reviews in Food Science and Nutrition* **2009**, *50* (1), 20–52. https://doi.org/10.1080/10408390802248585.

(10) BioSTEAM Development Group. Triacetic Acid Lactone Biorefineries, 2025. https://github.com/BioSTEAMDevelopmentGroup/Bioindustrial-Park/tree/master/biorefineries/TAL (accessed 08-18-2025).

(11) American Chemical Society. *CAS Registry Number 68-04-2: Sodium Citrate, Trisodium Citrate, C6H8O7.3Na. CAS SciFinder.*; Chemical Abstracts Service: Columbus, OH.

(12) Chia, M.; Schwartz, T. J.; Shanks, B. H.; Dumesic, J. A. Triacetic Acid Lactone as a Potential Biorenewable Platform Chemical. *Green Chemistry* **2012**, *14* (7), 1850–1853. https://doi.org/10.1039/c2gc35343a.

(13) Poling, B. E.; Prausnitz, J. M.; O'connell, J. P. *Properties of Gases and Liquids*, 5th ed.; McGraw-Hill Education, 2001; pp. 8.18, 8.185.

(14) Wohl, K. Thermodynamic Evaluation of Binary and Ternary Liquid Systems. *Trans. Am. Inst. Chem. Eng.* **1946**, *42*, 215–249.

(15) Dannenfelser, R.-M.; Yalkowsky, S. H. Estimation of Entropy of Melting from Molecular Structure: A Non-Group Contribution Method. *Ind. Eng. Chem. Res.* **1996**, *35* (4), 1483–1486. https://doi.org/10.1021/ie940581z.
(16) SciFinder; Chemical Abstracts Service: Columbus, OH. *Triacetic Acid Lactone; 2H-Pyran-2-One, 4-Hydroxy-6-Methyl-; RN 675-10-5.* https://scifinder.cas.org (accessed 2020-06-25).
(17) American Chemical Society. *CAS Registry Number: 7732-18-5, H2O, Water.* https://scifinder-n.cas.org/searchDetail/substance/655bc3286f952342635b662a/substanceDetails (accessed 2023-11-20).
(18) Fedors, R. F. A Method for Estimating Both the Solubility Parameters and Molar Volumes of Liquids. *Polym. Eng. Sci.* **1974**, *14* (2), 147–154. https://doi.org/10.1002/pen.760140211.
(19) Silberberg, M. S. *Principles of General Chemistry*, 1st ed.; 2007.
(20) CONGRESS, O. H. S. COMMITTEE ON WAYS AND MEANS US HOUSE OF REPRESENTATIVES. In *74th Congress, 1st Session, Hearings on HR*; 2018; Vol. 4120.
(21) Management, O. of; Staff, B. (US); others. *Analytical Perspectives: Budget of the US Government, Fiscal Year 2024*; Government Publishing Office, 2023.
(22) Watson, G.; The Tax Foundation. Trump Corporate Tax Rate Cut Proposal: Details & Analysis. https://taxfoundation.org/blog/trump-corporate-tax-cut/ (accessed 2023-11-20).
(23) Cortés-Peña, Y. R.; Kurambhatti, C.; Eilts, K.; Singh, V.; Guest, J. S. Economic and Environmental Sustainability of Vegetative Oil Extraction Strategies at Integrated Oilcane and Oil-Sorghum Biorefineries. *ACS Sustainable Chem. Eng.* **2022**, *10* (42), 13980–13990. https://doi.org/10.1021/acssuschemeng.2c04204.
(24) Huang, H.; Long, S.; Singh, V. Techno-Economic Analysis of Biodiesel and Ethanol Co-Production from Lipid-Producing Sugarcane. *Biofuels, Bioproducts and Biorefining* **2016**, *10* (3), 299–315. https://doi.org/10.1002/bbb.1640.
(25) Huang, H.; Long, S. P.; Clemente, T. E.; Singh, V. Technoeconomic Analysis of Biodiesel and Ethanol Production from Lipid-Producing Sugarcane and Sweet Sorghum. *Industrial Biotechnology* **2016**, *12* (6), 357–365. https://doi.org/10.1089/ind.2016.0013.
(26) Adom, F.; Dunn, J. B.; Han, J.; Sather, N. Life-Cycle Fossil Energy Consumption and Greenhouse Gas Emissions of Bioderived Chemicals and Their Conventional Counterparts. *Environ. Sci. Technol.* **2014**, *48* (24), 14624–14631. https://doi.org/10.1021/es503766e.
(27) Dunn, J. B.; Adom, F.; Sather, N.; Han, J.; Snyder, S.; He, C.; Gong, J.; Yue, D.; You, F. *Life-Cycle Analysis of Bioproducts and Their Conventional Counterparts in GREET*; ANL/ESD-14/9 Rev.; Argonne National Lab. (ANL), Argonne, IL (United States), 2015. https://doi.org/10.2172/1250468.
(28) WG, I. The Physical Science Basis. *Contribution of working group I to the fifth assessment report of the intergovernmental panel on climate change* **2013**, *1535*.
(29) Office of Energy Efficiency & Renewable Energy; U.S. Department of Energy. *Guidelines to Determine Life Cycle Greenhouse Gas Emissions of Clean Transportation Fuel Production Pathways Using 45ZCF-GREET*; 2025. https://www.energy.gov/sites/default/files/2025-05/45zcf-greet-user-manual-may2025.pdf.
(30) Wernet, G., Bauer, C., Steubing, B., Reinhard, J., Moreno-Ruiz, E., and Weidema, B.,. The Ecoinvent Database Version 3 (Part I): Overview and Methodology. *The International Journal of Life Cycle Assessment* **2016**, *21* (9), 1218–1230.
(31) U.S. EPA. *Lifecycle Analysis of Greenhouse Gas Emissions under the Renewable Fuel Standard*. https://www.epa.gov/renewable-fuel-standard-program/lifecycle-analysis-greenhouse-gas-emissions-under-renewable-fuel (accessed 2022-06-17).
(32) Argonne National Laboratory. GREET 2020 Model. October 10, 2020.
(33) Li, Y.; Bhagwat, S. S.; Cortés-Peña, Y. R.; Ki, D.; Rao, C. V.; Jin, Y.-S.; Guest, J. S. Sustainable Lactic Acid Production from Lignocellulosic Biomass. *ACS Sustainable Chem. Eng.* **2021**, *9* (3), 1341–1351. https://doi.org/10.1021/acssuschemeng.0c08055.

(34) Bhagwat, S. S.; Li, Y.; Cortés-Peña, Y. R.; Brace, E. C.; Martin, T. A.; Zhao, H.; Guest, J. S. Sustainable Production of Acrylic Acid via 3-Hydroxypropionic Acid from Lignocellulosic Biomass. *ACS Sustainable Chem. Eng.* **2021**, *9* (49), 16659–16669. https://doi.org/10.1021/acssuschemeng.1c05441.
(35) Tran, V. G.; Mishra, S.; Bhagwat, S. S.; Shafaei, S.; Shen, Y.; Allen, J. L.; Crosly, B. A.; Tan, S.-I.; Fatma, Z.; Rabinowitz, J.; Guest, J. S.; Singh, V.; Zhao, H. *An End-to-End Pipeline for Succinic Acid Production at an Industrially Relevant Scale Using* Issatchenkia Orientalis; preprint; Synthetic Biology, 2023. https://doi.org/10.1101/2023.04.30.538856.
(36) Junqueira, T. L.; Chagas, M. F.; Gouveia, V. L. R.; Rezende, M. C. A. F.; Watanabe, M. D. B.; Jesus, C. D. F.; Cavalett, O.; Milanez, A. Y.; Bonomi, A. Techno-Economic Analysis and Climate Change Impacts of Sugarcane Biorefineries Considering Different Time Horizons. *Biotechnology for Biofuels* **2017**, *10* (1), 50. https://doi.org/10.1186/s13068-017-0722-3.
(37) Markham, K. A.; Palmer, C. M.; Chwatko, M.; Wagner, J. M.; Murray, C.; Vazquez, S.; Swaminathan, A.; Chakravarty, I.; Lynd, N. A.; Alper, H. S. Rewiring Yarrowia Lipolytica toward Triacetic Acid Lactone for Materials Generation. *PNAS* **2018**, *115* (9), 2096–2101. https://doi.org/10.1073/pnas.1721203115.
(38) Wu, M.; Di, J.; Gong, L.; He, Y.-C.; Ma, C.; Deng, Y. Enhanced Adipic Acid Production from Sugarcane Bagasse by a Rapid Room Temperature Pretreatment. *Chemical Engineering Journal* **2023**, *452*, 139320. https://doi.org/10.1016/j.cej.2022.139320.
(39) Zhao, M.; Huang, D.; Zhang, X.; Koffas, M. A. G.; Zhou, J.; Deng, Y. Metabolic Engineering of Escherichia Coli for Producing Adipic Acid through the Reverse Adipate-Degradation Pathway. *Metabolic Engineering* **2018**, *47*, 254–262. https://doi.org/10.1016/j.ymben.2018.04.002.
(40) Demarteau, J.; Cousineau, B.; Wang, Z.; Bose, B.; Cheong, S.; Lan, G.; Baral, N. R.; Teat, S. J.; Scown, C. D.; Keasling, J. D.; Helms, B. A. Biorenewable and Circular Polydiketoenamine Plastics. *Nature Sustainability* **2023**. https://doi.org/10.1038/s41893-023-01160-2.
(41) Transparency Market Research. *Sorbic Acid Market: Global Industry Analysis, Size, Share, Growth, Trends, and Forecast, 2019–2030*; 2020.
(42) Chia, M.; Schwartz, T. J.; Shanks, B. H.; Dumesic, J. A. Triacetic Acid Lactone as a Potential Biorenewable Platform Chemical. *Green Chem.* **2012**, *14* (7), 1850–1853. https://doi.org/10.1039/C2GC35343A.
(43) Kim, M. S.; Choi, D.; Ha, J.; Choi, K.; Yu, J.-H.; Dumesic, J. A.; Huber, G. W. Catalytic Strategy for Conversion of Triacetic Acid Lactone to Potassium Sorbate. *ACS Catal.* **2023**, 14031–14041. https://doi.org/10.1021/acscatal.3c02775.
(44) Yu, J.; Landberg, J.; Shavarebi, F.; Bilanchone, V.; Okerlund, A.; Wanninayake, U.; Zhao, L.; Kraus, G.; Sandmeyer, S. Bioengineering Triacetic Acid Lactone Production in Yarrowia Lipolytica for Pogostone Synthesis. *Biotechnology and Bioengineering* **2018**, *115* (9), 2383–2388. https://doi.org/10.1002/bit.26733.
(45) Wang, Y.; Bao, R.; Huang, S.; Tang, Y. Bioinspired Total Synthesis of Katsumadain A by Organocatalytic Enantioselective 1,4-Conjugate Addition. *Beilstein J. Org. Chem.* **2013**, *9*, 1601–1606. https://doi.org/10.3762/bjoc.9.182.
(46) Song, L.; Yao, H.; Zhu, L.; Tong, R. Asymmetric Total Syntheses of (−)-Penicipyrone and (−)-Tenuipyrone via Biomimetic Cascade Intermolecular Michael Addition/Cycloketalization. *Org. Lett.* **2013**, *15* (1), 6–9. https://doi.org/10.1021/ol303071t.
(47) Chia, M.; Haider, M. A.; Pollock, G.; Kraus, G. A.; Neurock, M.; Dumesic, J. A. Mechanistic Insights into Ring-Opening and Decarboxylation of 2-Pyrones in Liquid Water and Tetrahydrofuran. *Journal of the American Chemical Society* **2013**, *135* (15), 5699–5708. https://doi.org/10.1021/ja312075r.
(48) CHEMANALYST. *Sorbic Acid Market Analysis: Industry Market Size, Plant Capacity, Production, Operating Efficiency, Demand & Supply Gap, End-User Industries, Sales Channel, Regional Demand, Company Share, Manufacturing Process, 2015-2034*; 2024. https://www.chemanalyst.com/industry-report/sorbic-acid-market-3061# (accessed 2024-05-28).

(49) Alibaba.com. *Lifecare Supply Potassium Sorbate High Quality Potassium Sorbate Granular - Shaanxi Lifecare Biotechnology Co., Ltd*. https://www.alibaba.com/product-detail/Lifecare-Supply-Potassium-Sorbate-High-Quality_1600897125355.html (accessed 2023-11-20).
(50) Cortés-Peña, Y. R.; Kurambhatti, C.; Eilts, K.; Singh, V.; Guest, J. S. Economic and Environmental Sustainability of Vegetative Oil Extraction Strategies at Integrated Oilcane and Oil-Sorghum Biorefineries. *ACS Sustainable Chem. Eng.* **2022**, *10* (42), 13980–13990. https://doi.org/10.1021/acssuschemeng.2c04204.
(51) Li, Y.; Kontos, G. A.; Cabrera, D. V.; Avila, N. M.; Parkinson, T. W.; Viswanathan, M. B.; Singh, V.; Altpeter, F.; Labatut, R. A.; Guest, J. S. Design of a High-Rate Wastewater Treatment Process for Energy and Water Recovery at Biorefineries. *ACS Sustainable Chem. Eng.* **2023**, *11* (9), 3861–3872. https://doi.org/10.1021/acssuschemeng.2c07139.
(52) Tang, S.-Y.; Qian, S.; Akinterinwa, O.; Frei, C. S.; Gredell, J. A.; Cirino, P. C. Screening for Enhanced Triacetic Acid Lactone Production by Recombinant Escherichia Coli Expressing a Designed Triacetic Acid Lactone Reporter. *Journal of the American Chemical Society* **2013**, *135* (27), 10099–10103. https://doi.org/10.1021/ja402654z.
(53) Xie, D.; Shao, Z.; Achkar, J.; Zha, W.; Frost, J. W.; Zhao, H. Microbial Synthesis of Triacetic Acid Lactone. *Biotechnology and Bioengineering* **2006**, *93* (4), 727–736. https://doi.org/10.1002/bit.20759.
(54) Cao, M.; Tran, V. G.; Qin, J.; Olson, A.; Mishra, S.; Schultz, J.; Huang, C.; Xie, D.; Zhao, H. Metabolic Engineering of Oleaginous Yeast Rhodotorula Toruloides for Overproduction of Triacetic Acid Lactone. *Biotechnology and Bioengineering* **2022**, *119* (9), 2529–2540. https://doi.org/10.1002/bit.28159.
(55) Cardenas, J.; Da Silva, N. A. Metabolic Engineering of Saccharomyces Cerevisiae for the Production of Triacetic Acid Lactone. *Metabolic Engineering* **2014**, *25*, 194–203. https://doi.org/10.1016/j.ymben.2014.07.008.
(56) Cardenas, J.; Da Silva, N. A. Engineering Cofactor and Transport Mechanisms in Saccharomyces Cerevisiae for Enhanced Acetyl-CoA and Polyketide Biosynthesis. *Metabolic Engineering* **2016**, *36*, 80–89. https://doi.org/10.1016/j.ymben.2016.02.009.
(57) Saunders, L. P.; Bowman, M. J.; Mertens, J. A.; Da Silva, N. A.; Hector, R. E. Triacetic Acid Lactone Production in Industrial Saccharomyces Yeast Strains. *Journal of Industrial Microbiology and Biotechnology* **2015**, *42* (5), 711–721. https://doi.org/10.1007/s10295-015-1596-7.
(58) Sun, L.; Lee, J. W.; Yook, S.; Lane, S.; Sun, Z.; Kim, S. R.; Jin, Y.-S. Complete and Efficient Conversion of Plant Cell Wall Hemicellulose into High-Value Bioproducts by Engineered Yeast. *Nature Communications* **2021**, *12* (1). https://doi.org/10.1038/s41467-021-25241-y.
(59) Liu, H.; Marsafari, M.; Wang, F.; Deng, L.; Xu, P. Engineering Acetyl-CoA Metabolic Shortcut for Eco-Friendly Production of Polyketides Triacetic Acid Lactone in Yarrowia Lipolytica. *Metabolic Engineering* **2019**, *56*, 60–68. https://doi.org/10.1016/j.ymben.2019.08.017.
(60) U.S. Energy Information Administration. *Annual Energy Outlook*. https://www.eia.gov/outlooks/aeo/ (accessed 2019-05-22).
(61) ICIS. *Chemical Market Reporter*; 2008. https://web.archive.org/web/20161125084558/http://www.icis.com:80/chemicals/channel-info-chemicals-a-z/ (accessed 2024-05-22).
(62) Cortes-Peña, Y.; Kumar, D.; Singh, V.; Guest, J. S. BioSTEAM: A Fast and Flexible Platform for the Design, Simulation, and Techno-Economic Analysis of Biorefineries under Uncertainty. *ACS Sustainable Chem. Eng.* **2020**, *8* (8), 3302–3310. https://doi.org/10.1021/acssuschemeng.9b07040.
(63) Humbird, D.; Davis, R.; Tao, L.; Kinchin, C.; Hsu, D.; Aden, A.; Schoen, P.; Lukas, J.; Olthof, B.; Worley, M.; Sexton, D.; Dudgeon, D. *Process Design and Economics for Biochemical Conversion of Lignocellulosic Biomass to Ethanol: Dilute-Acid Pretreatment and Enzymatic*

Hydrolysis of Corn Stover; Technical Report NREL/TP-5100-47764; National Renewable Energy Lab (NREL), 2011. http://www.nrel.gov/docs/fy11osti/47764.pdf (accessed 2015-09-13).
(64) Davis, R. E.; Grundl, N. J.; Tao, L.; Biddy, M. J.; Tan, E. C.; Beckham, G. T.; Humbird, D.; Thompson, D. N.; Roni, M. S. *Process Design and Economics for the Conversion of Lignocellulosic Biomass to Hydrocarbon Fuels and Coproducts: 2018 Biochemical Design Case Update*; NREL/TP-5100-71949; NREL, 2018. https://doi.org/10.2172/1483234.
